# Application of the TaqMan ARMS-PCR Approach for Genotyping Drug-Induced Hearing Loss Using Dried Blood Samples

**DOI:** 10.3390/cimb46060326

**Published:** 2024-05-29

**Authors:** Jiefeng Tan, Xiaoqing Zhang, Xue Wei, Min Ding

**Affiliations:** Key Laboratory of Clinical Laboratory Diagnostics, Ministry of Education of China, School of Laboratory Medicine, Chongqing Medical University, Chongqing 400016, China; jelftan@163.com (J.T.); xzhang5@cqmu.edu.cn (X.Z.); 2022110551@stu.cqmu.edu.cn (X.W.)

**Keywords:** amplification refractory mutation system, nucleic acid detection, genotyping, deafness, single nucleotide polymorphism

## Abstract

A single nucleotide variant in mitochondrial DNA (mtDNA) 1555A>G is associated with drug-induced hearing loss. For the 1555A>G mutation site, 1555A wild-type and 1555G mutant-type plasmids were constructed, respectively. In this study, a PCR method based on the TaqMan amplification refractory mutation system was proposed to detect mtDNA 1555A>G. A common upstream primer, a common TaqMan probe, and two downstream allele-specific primers with mismatched bases were designed. One-step amplification and detection of the wild-type and mutant type at the 1555 site were realized for the deafness-related gene through two reactions. Based on this detection method, the minimum detection limit of the wild-type and mutant type detection systems for plasmids was 50 copies/μL. The minimum sensitivity for the detection of nucleic acids in real dried blood spot (DBS) samples was 0.1 ng/μL. In the normal DBS DNA sample, the detection limit of the mutation abundance reached 0.78%. The specificity of the detection method was 100%, and the coefficient of variation was less than 3.36%. This approach was validated using clinical DNA extracted from 113 DBS samples of newborns. Additionally, it showed 100% agreement with bi-directional Sanger sequencing. It can be used as an optional method for the clinical detection of deafness-related genes.

## 1. Introduction

The World Health Organization (WHO) report in 2021, stated that there are approximately 430 million deaf individuals globally, and this number is estimated to increase to 710 million by 2050 [1]. Deafness is usually caused by both genetic and environmental factors, with about 50% attributed to genetic factors [2]. It can be categorized into autosomal dominant inheritance, autosomal recessive inheritance, X-linked inheritance, and mitochondrial inheritance. Among the genetic causes of deafness, mutations in mtDNA are closely associated with aminoglycoside-induced deafness and non-syndromic deafness. Studies have shown that the 1555A>G and 1494C>T mutations in mtDNA are the genetic basis for maternally inherited aminoglycoside-induced deafness, with aminoglycoside antibiotics being the main cause of deafness [3,4]. The prevalence of mtDNA mutations varies among different regions worldwide, with the occurrence rate of the 1555A>G mutation ranging from 0.08% to 0.7% in the general population and from 0.42% to 17% in the deaf population. The occurrence rates for the 1494C>T mutation in the general and deaf population are 0.01–0.25% and 0.18–0.7%, respectively [5]. It is evident that the 1555A>G mutation is the most common mutation associated with aminoglycoside-induced deafness, whereas the 1494C>T mutation is relatively rare.

Aminoglycoside antibiotics have strong antibacterial effects against Gram-negative bacilli and Mycobacterium tuberculosis [6]. In developed countries, aminoglycoside antibiotics are primarily used to treat hospitalized patients with Gram-negative bacterial infections, such as tuberculosis and cystic fibrosis. In many developing countries, the use of aminoglycoside antibiotics is more widespread, and they are even used to control mild infections in the absence of effective monitoring methods [7]. Currently, effective treatment measures are still insufficient for aminoglycoside-induced deafness. Therefore, the prevention of aminoglycoside-induced deafness is of utmost importance [8]. Pre-marital genetic screening and screening for deafness-related genes in pregnant women and newborns can enable early detection and intervention. For high-risk individuals, such as children sensitive to aminoglycoside-induced deafness, the use of relevant aminoglycoside antibiotics should be avoided or reduced.

Currently, genetic testing technologies for deafness include new techniques, such as restriction fragment length polymorphism (RFLP) [9], copy number variation sequencing (CNV-Seq) [10], whole exome sequencing (WES) [11], next-generation sequencing (NGS) [12], multiplex ligation-dependent probe amplification (MLPA) [13], reverse dot blot (RDB) [14], denaturing high-performance liquid chromatography (dHPLC) [15], microarray gene chip detection technology [16], matrix-assisted laser desorption/ionization time of flight mass spectrometry (MALDI-TOF-MS) [17], high-resolution melting (HRM) [18], and Sanger sequencing. However, these technologies have disadvantages, such as high environmental requirements, complex experimental operations, relatively expensive equipment, and long testing times, making it difficult to popularize them in grassroots hospitals and community clinics.

The amplification refractory mutation system (ARMS) polymerase chain reaction (PCR) utilizes the principle that if the 3′ end base of a primer does not complement the template base, the extension will not occur [19]. This system enables discrimination between the templates with point mutations and normal templates by ensuring that the 3′ end base of the primer specifically complements either the mutation or the wild-type template base. ARMS-PCR can detect single nucleotide mutations and small deletions on DNA molecules simply by introducing mismatched bases in the primer design [20]. This method has wide applications in genetic analysis, mutation identification, and human leukocyte antigen (HLA) typing [21]. The specificity of the ARMS-PCR gene typing method relies on the properties of DNA polymerase, reaction conditions, and the nature of the ARMS primers themselves. In clinical applications, introducing mismatched bases only at the 3′ end may not entirely inhibit extension reactions; thus, mismatched bases are artificially introduced two to three bases away from the 3′ end of the primer to further enhance specificity [22].

The TaqMan probe technique has the advantages of low equipment cost, simple operation, and short processing time. Moreover, it provides high specificity by precisely targeting the DNA sequence of interest [23,24]. The TaqMan-ARMS-PCR technology is based on a real-time fluorescence quantitative PCR platform, utilizing ARMS primers for specific PCR amplification of the mutation target sequence and TaqMan probes for specific site detection of the amplified products, thereby genotyping the gene. We utilized a common upstream primer, a common TaqMan probe, and two downstream allele-specific primers to establish a TaqMan-ARMS-PCR system for genotyping 1555A>G mutation in the DNA extracted from clinical DBS samples. Gene typing was determined based on the fluorescence curve, making it suitable for the rapid screening of deafness-related genes.

## 2. Materials and Methods

### 2.1. Plasmid Construction and Preparation of the Real Sample

To obtain the standard template, two plasmids containing the 1555A and 1555G alleles were constructed separately. Plasmid construction and verification via sequencing were completed by Sangon Biotech Co., Ltd. (Shanghai, China) (Figures S1 and S2, Table S1). Then, the recombined plasmids were purified using a Plasmid Purification Kit by Bioeasun Biotech Co., Ltd. (Guangzhou, China). The final DNA quality and concentration were measured using the NanoDrop UV-Vis spectrophotometer (Thermo Fisher Scientific Corporation, Waltham, MA, USA).

This study enrolled 113 newborn infants from October 2020 to April 2023 in the First Affiliated Hospital of Chongqing Medical University. Signed consent was obtained from the parents of the newborns. Ethical approval was obtained from the Ethics Committee of First Affiliated Hospital of Chongqing Medical University. Three drops of blood from a heel prick were placed on an FTA™ Indicated Micro Card (Whatman, UK). The specimens were air dried for 4 h and transferred to the laboratory. The specimens were refrigerated between 2 °C and 10 °C. For DNA extraction, three 3 mm punches were collected from each DBS into a 2 mL tube, and the extraction was performed using the superparamagnetic-bead-based FTA^TM^ DNA Extraction Kit manufactured by Bioeasun Biotech Co., Ltd. (Guangzhou, China). The extraction was carried out according to the manufacturer’s instructions for the FTA. The standard superparamagnetic-bead based DNA extraction protocol includes five principal steps: the digestion step, the lysis step, binding to magnetic-beads, washing, and elution. 

### 2.2. Primer and Probe Design and TaqMan-ARMS-PCR Detection

**Figure 1:**
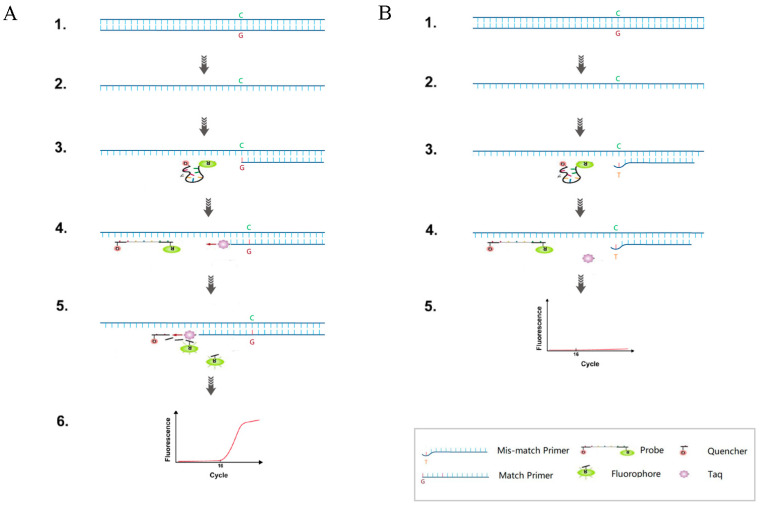
TaqMan-ARMS-PCR diagram. (**A**) Base match and (**B**) base mismatch.

Specific primers for the wild-type and mutant sites, as well as common upstream primers and a common probe, were designed using Primer Premier 6.0 software. The secondary structures were analyzed using Oligo7 software and their specificity was confirmed by comparing each sequence through BLAST. The design was based on the MT-RNR1 (12S rRNA, GenBank accession number: ID4549) gene sequence (Table S2). The TaqMan-ARMS-PCR for single nucleotide polymorphism (SNP) genotyping requires setting up two reaction systems for amplification of the same sample. A set of shared upstream primers, a shared probe, and two allele-specific downstream primers were designed to distinguish between wild-type and mutant alleles, as shown in Figure 1. The 3′ end of the specific primers completely matched the SNP site. If there was a mismatch between the primer’s 3′ end base and the non-complementary template, the formation of the phosphodiester bond was hindered, preventing further extension. To increase specificity and enhance discrimination between the two gene types, an additional mismatch base at the third position from the 3′ end of the specific primers was introduced to hinder the extension of incompletely matched primers. The common TaqMan probe for detecting the 1555A>G allele was labeled with an FAM fluorescent reporter group at the 5′ end and a BHQ1 fluorescent quencher group at the 3′ end.

**Figure 2:**
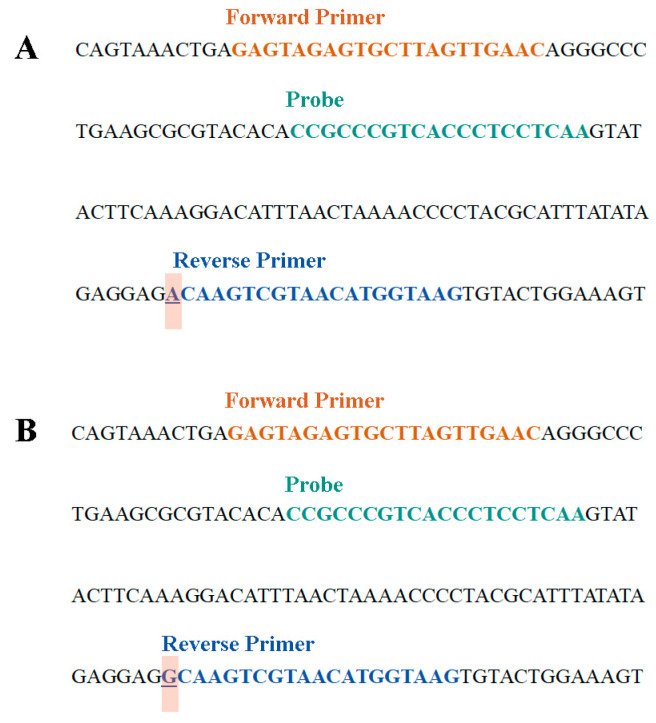
Primers and probe design location. (**A**) 1555A detection system and (**B**) 1555G detection system.

The primers and probes used are as follows (5′-3′): Forward primer, GAGTAGAGTGCTTAGTTGAAC; wild-reverse primer, CTTACCATGTTACGACTAGT; and mutant-reverse primer, CTTACCATGTTACGACTCGC; Probe, FAM-CCGCCCGTCACCCTCCTCAA-BHQ-1. The location of the nucleotides are shown in Figure 2. All primer and probe sequences used in this research were synthesized by Sangon Biotech (Shanghai, China). 

### 2.3. TaqMan-ARMS-PCR Reaction

The TaqMan-ARMS-PCR reaction was carried out in 20 μL of the reaction volume. The reaction mixture contained 4 µL of the 5× SNUPP Taq Buffer, 0.4 µL of the SNUPP DNA Taq (5 U/μL) (Turtle-tech, Shanghai, China), 0.4 µL of the forward primer (10 μM), 0.4 µL of the reverse primer (10 μM), 0.01 µL of the probe (10 μM), 5 µL of the template DNA, and the rest was made up with DEPC-treated water. Each DNA sample was amplified by two sets of primer–probes in reaction tubes separately. The wild-type set amplification used the forward primer, the wild-reverse primer, and a probe. The mutant set amplification used the forward primer, the mutant-reverse primer, and a probe. The final results were analyzed using an amplification curve. The reactions were performed in a SLAN-96P fluorescence qPCR detection system (Hongshi Biotech, Shanghai, China). After the PCR procedure was optimized, the procedure used for the TaqMan-ARMS-PCR assay for 1555A>G was as follows: predenaturation at 95 °C for 150 s, followed by 28 cycles with denaturation at 95 °C for 15 s, annealing at 48 °C for 40 s, extension at 68 °C for 60 s, and at the end of each cycle, the FAM fluorescence signal was collected using the End Points mode and was detected. The analysis was conducted utilizing the proprietary analysis software V8.2.2 of the SLAN-96P system. 

### 2.4. Analysis of the Sensitivity, Specificity, and Repeatability

A series of ratios of mutant-type plasmid were prepared (100%, 50%, 25%, 12.5%, 6.25%, 3.13%, 1.56%, 0.78%, and 0%) to evaluate the sensitivity of the TaqMan-ARMS-PCR assay. The specificity of the assay was measured using the different types of plasmids (1555A, 1555G, 1555A/G, and H_2_O). The assay’s intra-day and inter-day precision were also evaluated with three different concentrations (5.0 × 10^4^ copies/μL, 5.0 × 10^3^ copies/μL, and 5.0 × 10^2^ copies/μL) of wild-type and mutant plasmid standards individually. Three replicates were set for each sample concentration. 

### 2.5. The Detection Limit of Plasmid and Real Samples

A series of ratios of plasmid were prepared (5 copies/μL, 5 × 10^1^ copies/μL, 5.0 × 10^2^ copies/μL, 5.0 × 10^3^ copies/μL, 5.0 × 10^4^ copies/μL, and 5.0 × 10^5^ copies/μL) to evaluate the detection limit of plasmid of the TaqMan-ARMS-PCR assay. A series of ratios of real DNA samples were prepared (10 ng/μL, 5 ng/μL, 1 ng/μL, 0.5 ng/μL, 0.25 ng/μL, and 0.1 ng/μL) to evaluate the detection limit of real samples for the assay.

### 2.6. Genotyping Validation

A total of 113 clinical samples were tested using bi-directional Sanger sequencing, which is a gold standard method for detecting DNA mutations [25]. The TaqMan-ARMS-PCR assay results were compared with the results from DNA sequencing. The primers used for Sanger sequencing are as follows (5′-3′): forward primer, GAGGTGGCAAGAAATGGG and reverse primer, GGTAAATGGTTTGGCTAAGGT. The Sanger sequencing PCR reaction was carried out in 25 μL of the reaction volume. The reaction mixture contained 5 µL of the 5× Anstart Taq buffer (Fapon Biotech Inc., Shenzhen, China), 0.3 µL of dNTP (100 mM) (Abclonal, Shanghai, China), 0.25 µL of the forward primer (50 μM), 0.25 µL of the reverse primer (50 μM), 0.25 µL of Anstart Taq (5 U/μL) (Fapon Biotech Inc., Shenzhen, China), 10 µL of the template DNA, and the rest was made up with DEPC-treated water. The reactions were performed in a SLAN-96P fluorescence qPCR detection system (Hongshi, Shanghai, China). The PCR procedure was as follows: incubated at 50 °C for 120 s, predenaturalized at 95 °C for 150 s, followed by 45 cycles with denaturation at 95 °C for 15 s, and extension at 60 °C for 30 s. The PCR product was then sequenced by the local merchant (Sangon Biotech, Shanghai, China). 

### 2.7. Statistical Analysis

The concordance rates of the test results obtained using direct sequencing of the PCR products and the TaqMan-ARMS-PCR assay were compared using the Kappa concordance test and SPSS Statistics V20.0 (SPSS, Inc., Chicago, IL, USA). A *p* value of less than 0.05 was considered statistically significant. Graphs were generated using OriginPro software V2018 (Northampton, MA, USA).

## 3. Results

### 3.1. Optimization of TaqMan-ARMS-PCR

**Figure 3:**
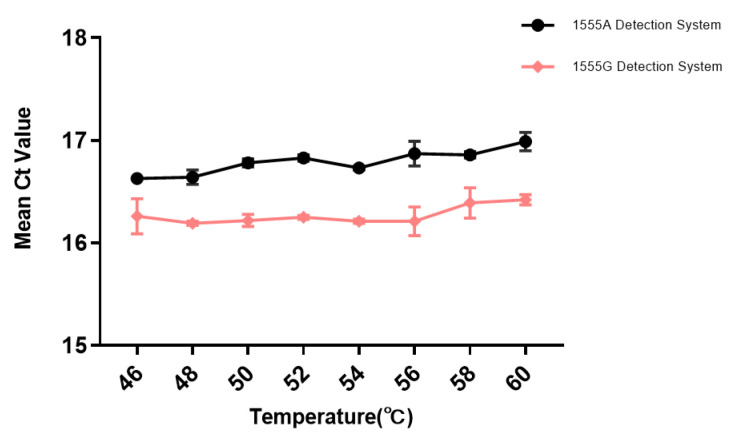
Ct value in 1555A/G plasmid TaqMan-ARMS-PCR detection for different annealing temperatures (*n* = 3).

The annealing temperature is one of the important influencing factors in PCR amplification systems, which has a significant impact on the amount and specificity of amplification products. Low annealing temperatures can result in the non-specific binding of primers and templates, leading to false-positive results. Conversely, high temperatures can reduce the binding between the template and primers, thereby decreasing the amplification efficiency. In order to obtain the optimal amplification efficiency, annealing temperatures were set at 46 °C, 48 °C, 50 °C, 52 °C, 54 °C, 56 °C, 58 °C, and 60 °C, on the basis of the reaction system. A total of 5.0 × 10^3^ copies/μL of wild-type plasmid and mutant plasmid were added to both the wild-type and mutant groups for each of the three parallel experiments to determine the most suitable annealing temperature. According to the detection results of the amplification curves at different temperatures, both the wild-type and mutant detection systems achieved detection of the corresponding genes within the range of 46 °C to 60 °C, as shown in Figure 3. At the same annealing temperature, the cycle threshold (Ct) of the mutant detection system was lower than that of the wild-type detection system, with a difference within 0.5 Ct. The Ct values of both detection systems increased with increasing annealing temperature, with a change of within 1 Ct. When the annealing temperature was 48 °C, the Ct values of the amplification curves for both the wild-type and mutant groups were the smallest, indicating the highest amplification efficiency. Therefore, 48 °C was selected as the optimal annealing temperature for TaqMan-ARMS-PCR.

**Figure 4:**
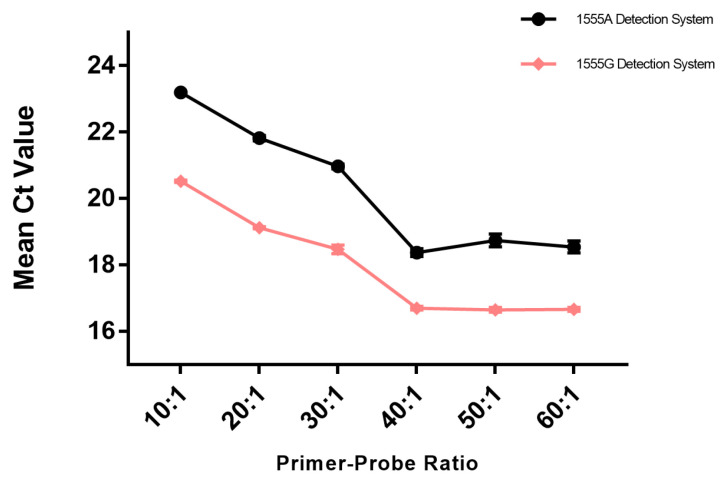
Ct value in 1555A/G plasmid TaqMan-ARMS-PCR detection for different primer–probe ratios (*n* = 3).

Another critical factor involved in the specificity and efficiency of ARMS-PCR was the primer–probe ratios. To achieve the best amplification efficiency with the optimized reaction system, the primer–probe ratios were set at 10:1, 20:1, 30:1, 40:1, 50:1, and 60:1. A total of 5.0 × 10^3^ copies/μL of wild-type plasmid and mutant plasmid were added to both the wild-type and mutant groups for each of the three parallel experiments to determine the optimal primer–probe ratio. Different primer–probe ratios were tested. Within the range of 10:1 to 60:1, both the wild-type and mutant detection systems could achieve detection of the corresponding genes, as shown in Figure 4. At the same primer–probe ratio, the Ct value of the mutant detection system was lower than that of the wild-type detection system, with a difference within 1.5. Within the range of 10:1 to 40:1, the Ct values of both detection systems decreased with an increasing primer–probe ratio, but after the ratio exceeded 40, the Ct value change tended to stabilize. Therefore, when the primer–probe ratio was 40:1, the Ct values of the amplification curves for both the wild-type and mutant groups were the smallest, indicating the highest amplification efficiency. Therefore, the optimal primer–probe ratio was set at 40:1 for TaqMan-ARMS-PCR.

### 3.2. Performance of the Analysis

**Figure 5:**
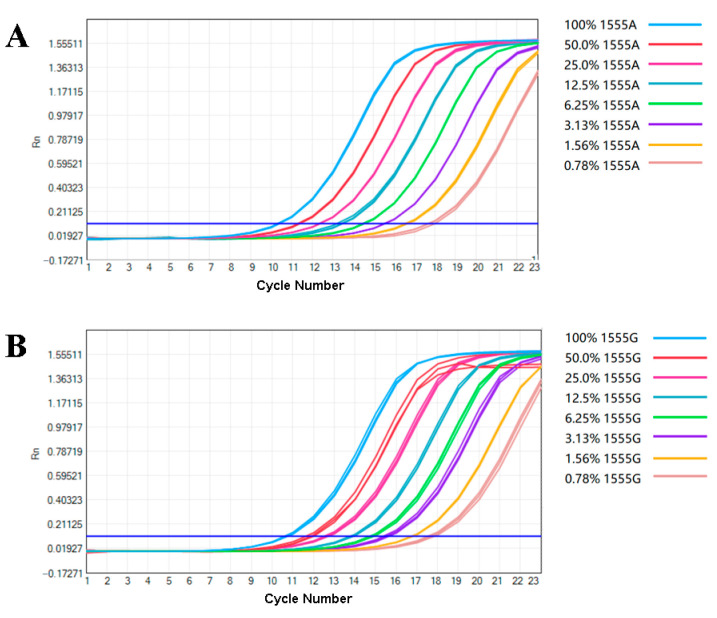
Amplification plots of detection systems with various wild/mutant plasmid ratios (*n* = 3). (**A**) 1555A detection system and (**B**) 1555G detection system.

Although SNP detection has traditionally been considered primarily qualitative, the use of inadequately processed samples can present challenges for this method. Consequently, a highly sensitive approach is preferable for accurate SNP detection. To evaluate the sensitivity of the TaqMan-ARMS-PCR assay for detecting samples with different mutation ratios, the wild-type and mutant plasmids with a concentration of 5.0 × 10^5^ copies/μL were mixed in different proportions to construct a series of standard samples with different loads: 100%, 50%, 25%, 12.5%, 6.25%, 3.13%, 1.56%, 0.78%, and 0%. Amplification was performed using the optimized reaction system, with each group subjected to three parallel experiments. As the concentration of the load standard samples decreased, the Ct values of the amplification curves increased, as shown in Figure 5 and Figure S3. When the content was 0, no amplification curves were observed for both amplification systems, and the Ct value was recorded as NoCt. Meanwhile, both the wild-type detection system and the mutant detection system could detect positive standard samples corresponding to 0.78~100%, indicating that besides detecting gene homozygotes, the TaqMan-ARMS-PCR detection method also meets the detection requirements of samples with heterozygous nucleic acids lower than 0.78%.

**Figure 6:**
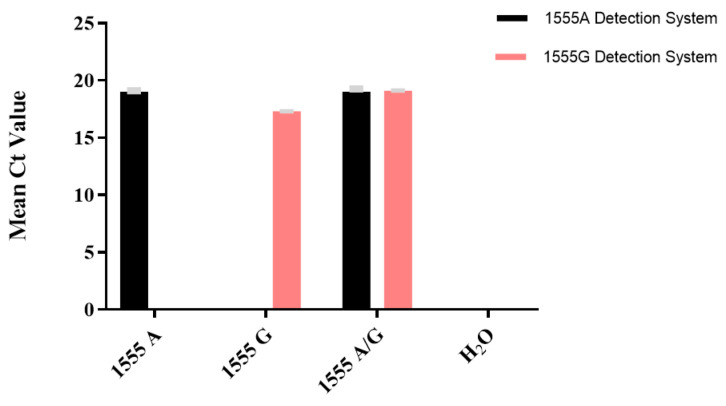
Specificity validation of 1555A/G plasmids using TaqMan-ARMS-PCR detection (*n* = 3).

For the mtDNA 1555A>G locus, the wild-type (1555A), mutant (1555G), heterozygous (1555A/G), and negative control (H_2_O) groups were designed to investigate the specificity of our method, as shown in Figure 6 and Figure S4. The wild-type detection system exhibited S-shaped amplification curves for heterozygous plasmids containing 1555A and 1555A/G, with Ct values below 20. No amplification curves were observed for plasmids containing 1555G and pure water, with Ct values recorded as NoCt. The mutant detection system displayed S-shaped amplification curves for heterozygous plasmids containing 1555G and 1555A/G, with Ct values below 20; however, no amplification curves were observed for plasmids containing wild-type 1555A and pure water, with Ct values recorded as NoCt. The specificity was 100%.

Using the wild-type detection system, the intra-day CV values for high, medium, and low concentrations of wild-type 1555A plasmids were 0.34%, 0.48%, and 0.47%, respectively, whereas the inter-day CV values were 3.36%, 2.67%, and 1.79%, respectively. Employing the mutant detection system, the intra-day CV values for high, medium, and low concentrations of mutant 1555G plasmids were 0.26%, 2.30%, and 2.63%, respectively, whereas the inter-day CV values were 1.53%, 1.64%, and 0.41%, respectively. The results, as shown in Table 1, indicate that the established TaqMan-ARMS-PCR method exhibits good precision.

### 3.3. Performance of the Detection Limit of Plasmid and Real Samples

The series of plasmid concentration standard solutions were separately amplified using the wild-type reaction system and the mutant reaction system. The results are shown in Figure S5. Within the range of 5.0 × 10^3^ copies/μL to 5.0 × 10^5^ copies/μL, the amplification curves exhibited three stages (a background fluorescence phase, an exponential amplification phase, and a plateau phase), showing a classic S-shaped curve. When the concentration was below 5.0 × 10^2^ copies/μL, only the amplification curve appeared without reaching a plateau phase.

**Figure 7:**
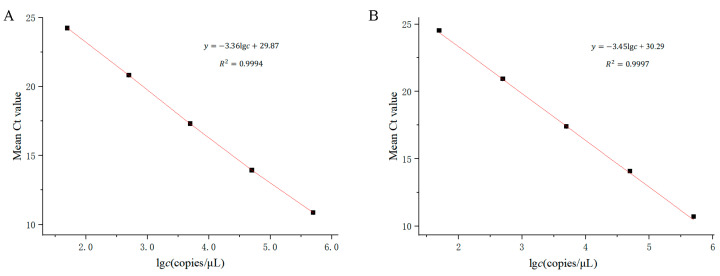
Calibration curves (*n* = 3). (**A**) 1555A detection system and (**B**) 1555G detection system.

Using the logarithm of plasmid concentration (*c*) as the horizontal axis and the average Ct value of the amplification curve (y) as the vertical axis, a linear regression analysis was conducted, as shown in Figure 7. The standard curve equation for wild-type detection was y = −3.36lg*c* + 29.87, with *R*^2^ = 0.9994. The linear range was from 50 copies/μL to 5 × 10^5^ copies/μL, with a minimum detection limit of 50 copies/μL. The standard curve equation for mutant detection was y = −3.45lg*c* + 30.29, with *R*^2^ = 0.9997. The linear range was also from 50 copies/μL to 5 × 10^5^ copies/μL, with a minimum detection limit of 50 copies/μL.

The series of concentration real sample nucleic acids were amplified using the wild-type reaction system and the mutant reaction system, and the results are shown in Figure S6. From the figure, it can be seen that within the range of 5 ng/μL to 10 ng/μL, the amplification curves exhibited three stages (a background fluorescence phase, an exponential amplification phase, and a plateau phase), showing a classic S-shaped curve. However, when the concentration was below 1 ng/μL, only the amplification curve appeared without reaching a plateau phase.

**Figure 8:**
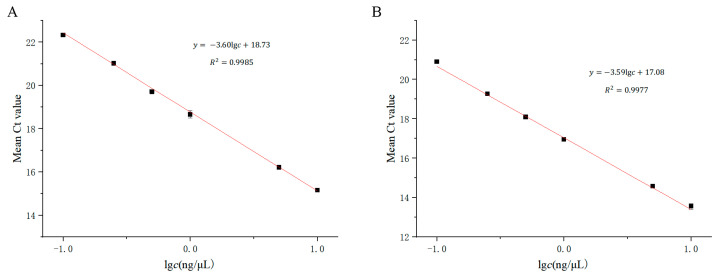
Linear regression analysis of serial dilution wild-type and mutant-type DBS DNA samples (*n* = 3). (**A**) 1555A detection system and (**B**) 1555G detection system.

Linear regression analysis was conducted using the logarithm of nucleic acid concentration (*c*) as the horizontal axis and the average Ct value of the amplification curve (y) as the vertical axis, as shown in Figure 8. The linear regression equation for wild-type detection was y = −3.60lg*c* + 18.73, with *R*^2^ = 0.9985. The linear range was from 0.1 ng/μL to 10 ng/μL, with a minimum detection limit of 0.1 ng/μL. The linear regression equation for mutant detection was y = −3.59lg*c* + 17.08, with *R*^2^ = 0.9977. The linear range was also from 0.1 ng/μL to 10 ng/μL, with a minimum detection limit of 0.1 ng/μL.

### 3.4. Performance of the TaqMan-ARMS-PCR Assay for Clinical Samples Compared with Sanger Sequencing

To evaluate the practical application of the TaqMan-ARMS-PCR assay to detect mtDNA 1555A>G, 113 DNA samples were assessed, and the results were compared with those obtained using bi-directional Sanger sequencing. The nucleic acid concentration of the collected DBS samples was 11.54 ± 5.43 ng/μL, with a mean value greater than 1 ng/μL. The agreement analysis, according to clinical sample detection, demonstrated that the Kappa values between the TaqMan-ARMS-PCR assay and Sanger sequencing were 1.0, *p* < 0.05 (Table 2 and Table S3). Additionally, in comparison to Sanger sequencing, the sensitivity of the TaqMan-ARMS-PCR assay was 100%, whereas the specificity of the TaqMan-ARMS-PCR assay was 100%. Thus, the TaqMan-ARMS-PCR assay showed perfect diagnostic agreement with Sanger sequencing to detect 1555A>G in clinical samples.

## 4. Discussion

Currently, the two common methods for gene mutation detection are Sanger sequencing and ARMS-PCR. Sanger sequencing, compared through forward and reverse bidirectional sequencing, is considered the “gold standard” for detecting gene mutations, but it is cumbersome to operate and time-consuming. Additionally, it has lower sensitivity and cannot detect gene mutations with a burden below 10% [26,27]; therefore, it has certain limitations. ARMS-PCR combines the advantages of Scorpions [28] and ARMS technologies, exhibiting significantly higher detection sensitivity than direct sequencing, and is capable of detecting mutations in samples with contents as low as 0.1% to 1.0%. However, traditional ARMS-PCR requires two or more pairs of primers [29], making it prone to primer dimer formation, which affects the overall detection performance. Moreover, the detection process involves agarose gel electrophoresis [30] and requires handling of strong carcinogens, such as ethidium bromide, posing certain hazards to operators. By combining ARMS-PCR with TaqMan probes, detection can be achieved using probes for amplicons, enabling real-time fluorescence quantitative PCR platforms to detect sample genetic polymorphisms. This detection process is simple and quick, requiring only 1 h to complete, effectively avoiding contact with ethidium bromide dye.

The key to improving the sensitivity and specificity of ARMS-PCR lies in the design of primers. In theory, Taq DNA polymerase can only perform effective amplification reactions when the 3′ end of the primer is completely complementary to the template base. However, due to the sensitivity of Taq DNA polymerase being influenced by multiple factors, even if the 3′ end base of the primer is not complementary to the template, extension can still occur. That is, if there is only one mismatched base at the 3′ end, the primer can still bind and extend, but its extension efficiency is lower than that of the primer with a matched 3′ end. Additionally, different mismatches at the 3′ end base have different extension efficiencies. Therefore, relying solely on one mismatched base at the 3′ end of the primer cannot reliably distinguish between two alleles, leading to false-positive results. Generally, introducing additional mismatched bases at the second or third position from the 3′ end, in conjunction with mismatched bases at the end, reduces the ability to form phosphodiester bonds at the 3′ end, significantly reducing the production of non-complementary amplification products [31]. This method introduces mismatched bases at the third position from the 3′ end of the downstream specific primer, inhibiting the detection ability of the wild-type detection system for mutant plasmids and the detection ability of the mutant detection system for wild-type plasmids. Combined with the action of the specific primer at the 3′ end, both systems exhibit good specificity.

While ensuring detection sensitivity, reducing the number of PCR amplification cycles can reduce the probability of false-positive results. Because of the presence of primers and Taq DNA polymerase, excessive cycling can cause non-specific amplification, leading to false-positive results. In this experiment, when the number of amplification cycles exceeded 30, non-specific amplification occurred. Therefore, considering the comprehensive detection sensitivity and experimental time, the number of cycles was set to 28, ensuring that no non-specific amplification occurred. Furthermore, the detection sensitivity reached 0.1 ng/μL, meeting the detection requirements. Choosing a DNA polymerase without 3′ → 5′ exonuclease activity is essential to ensure the function of ARMS-PCR. Without 3′ → 5′ exonuclease activity, it is impossible to correct 3′ end mismatches. When amplifying 3′ end mismatched primers, efficiency is significantly reduced. When the number or efficiency of mismatched bases reaches a certain level, the 3′ end base cannot be extended due to difficulty in forming phosphodiester bonds, resulting in termination of the reaction.

## 5. Conclusions

In this study, the specificity of detection was ensured from multiple dimensions by integrating the specificity of the TaqMan probe template, the specificity of ARMS-PCR primers, the optimization of PCR amplification cycles, and the selection of specific enzymes. By designing a shared upstream primer, a shared TaqMan probe, and two downstream allele-specific primers with mismatched bases, one-step amplification and detection of wild-type and mutant alleles at the 1555 locus of the deafness-related gene were achieved on a real-time fluorescence quantitative PCR platform. Both the wild-type and mutant detection systems demonstrated 100% specificity for different types of samples. Additionally, the plasmid detection sensitivity reached 50 copies/μL, the sensitivity of real sample nucleic acid was 0.1 ng/μL, and the mutation load detection limit was 0.78%, demonstrating good detection performance. It is of great significance to prevent drug-induced deafness through the genetic screening of drug-related deafness. So, the life quality of the patients can be greatly improved and the medical burden of families can be reduced. At the same time, compared with sequencing, this method solely requires a common real-time qPCR instrument, with the detection process achievable within a mere 1.5-h timeframe. Moreover, real-time qPCR machines are widely used in many countries, so the Taqman ARMS-PCR approach can be easily applied to perform SNP detection in pharmacogenomics and oncology.

## Figures and Tables

**Table 1:** The precision of the method.

Type	Conc. (Copies/μL)	Intra-Day (*n* = 10)		Inter-Day (*n* = 15)	
		Mean ± SD (Copies/μL)	CV (%)	Mean ± SD (Copies/μL)	CV (%)
Wild-type detection for 1555A plasmid	A-50000	14.19 ± 0.05	0.34%	14.17 ± 0.48	3.36%
	A-5000	17.47 ± 0.08	0.48%	17.51 ± 0.47	2.67%
	A-500	21.13 ± 0.10	0.47%	21.21 ± 0.38	1.79%
Mutant-type detection for 1555G plasmid	G-50000	14.28 ± 0.04	0.26%	14.28 ± 0.22	1.53%
	G-5000	17.49 ± 0.40	2.30%	17.63 ± 0.29	1.64%
	G-500	21.65 ± 0.57	2.63%	21.27 ± 0.09	0.41%

**Table 2:** Comparison of the test results between TaqMan-ARMS-PCR and Sanger sequencing (*n* = 113).

		Sanger Sequencing			Total
		1555AA	1555AG	1555GG	
TaqMan-ARMS-PCR	1555AA	100	0	0	100
	1555AG	0	6	0	6
	1555GG	0	0	7	7
	Total	100	6	7	113

## Data Availability

The data produced in this study are presented in this paper and in the Supplementary Materials.

## Supplementary Materials

The following supporting information can be downloaded at: https://www.mdpi.com/article/10.3390/cimb46060326/s1. Figure S1: Electropherograms of BamHI/SalI digestion. A: 1555A plasmid, B: 1555G plasmid; Figure S2: Sanger sequencing plots. A: 1555A plasmid, B: 1555G plasmid; Figure S3: Ct value of detection system with various wild/mutant plasmid ratios (*n* = 3). A: 1555A detection system, B: 1555G detection system; Figure S4: Amplification plots of 1555A/G plasmids (*n* = 3). A: 1555A detection system, B: 1555G detection system; Figure S5: Amplification plots of serial dilution wild and mutant plasmids (*n* = 3). A: 1555A detection system, B: 1555G detection system; Figure S6: Amplification plots of serial dilution wild-type and mutant-type DBS DNA samples (*n* = 3). A: 1555A detection system, B: 1555G detection system; Table S1: Plasmid sequences for the 1555A and 1555G genotypes; Table S2: Gene sequences of the 1555A and 1555G genotypes; Table S3: Detection of DBS DNA samples using ARMS-PCR and Sanger sequencing.
