# Supplementary material for: Application of the TaqMan ARMS-PCR Approach for Genotyping Drug-Induced Hearing Loss Using Dried Blood Samples
Source: Curr Issues Mol Biol. 2024 May 29;46(6):0. doi: 10.3390/cimb46060326 (PMC13176787; doi:10.3390/cimb46060326)
Supplement: Supplementary file 1 [file cimb-46-00326-s001.zip › Table S1.pdf]

Table S1 Plasmid sequences for the 1555A and 1555G genotypes

Name	Gene sequence (5’→3’)
	GTTCGTCCAAGTGCACTTTCCAGTACACTTACCATGTTACGACT
	TGTCTCCTCTATATAAATGCGTAGGGGTTTTAGTTAAATGTCCTT
1555A	TGAAGTATACTTGAGGAGGGTGACGGGCGGTGTGTACGCGCTT
Plasmid	CAGGGCCCTGTTCAACTAAGCACTCTACTCTTAGTTTACTGCTA
	AATCCACCTTCGACCCTTAAGTTTCATAAGGGCTATCGTAGTTT
	TCTGGGGTAGAAAATGTAGCCCATTTCTTGCCACCTCATGGGC
	GTTCGTCCAAGTGCACTTTCCAGTACACTTACCATGTTACGACT
	TGCCTCCTCTATATAAATGCGTAGGGGTTTTAGTTAAATGTCCTT
1555G	TGAAGTATACTTGAGGAGGGTGACGGGCGGTGTGTACGCGCTT
Plasmid	CAGGGCCCTGTTCAACTAAGCACTCTACTCTTAGTTTACTGCTA
	AATCCACCTTCGACCCTTAAGTTTCATAAGGGCTATCGTAGTTT
	TCTGGGGTAGAAAATGTAGCCCATTTCTTGCCACCTCATGGGC
