# Supplementary material for: Application of the TaqMan ARMS-PCR Approach for Genotyping Drug-Induced Hearing Loss Using Dried Blood Samples
Source: Curr Issues Mol Biol. 2024 May 29;46(6):0. doi: 10.3390/cimb46060326 (PMC13176787; doi:10.3390/cimb46060326)
Supplement: Supplementary file 1 [file cimb-46-00326-s001.zip › Table S2.pdf]

Table S2 Gene sequences of the 1555A and 1555G genotypes

Name	Gene sequence (5'→3')
1555A	AATAGGTTTGGTCCTAGCCTTTCTATTAGCTCTTAGTAAGAT<br>TACACATGCAAGCATCCCCGTTCCAGTGAGTTCACCCTCTAAATC<br>ACCACGATCAAAAGGAACAAGCATCAAGCACGCAGCAATGCAG<br>CTCAAAACGCTTAGCCTAGCCACACCCCCACGGGAAACAGCAGT<br>GATTAACCTTTAGCAATAAACGAAAGTTTAACTAAGCTATACTA<br>ACCCCAGGGTTGGTCAATTTCGTGCCAGCCACCGCGGTCACACG<br>ATTAACCCAAGTCAATAGAAGCCGGCGTAAAGAGTGTTTTAGAT<br>CACCCCCTCCCCAATAAAGCTAAAACTCACCTGAGTTGTAAAAA<br>ACTCCAGTTGACACAAAATAGACTACGAAAGTGGCTTTAACATA<br>TCTGAACACACAATAGCTAAGACCCAAACTGGGATTAGATACCC<br>CACTATGCTTAGCCCTAAACCTCAACAGTTAAATCAACAAAACT<br>GCTCGCCAGAACACTACGAGCCACAGCTTAAAACTCAAAGGACC<br>TGGCGGTGCTTCATATCCCTCTAGAGGAGCCTGTTCTGTAATCGA<br>TAAACCCCGATCAACCTCACCACCTCTTGCTCAGCCTATATACCG<br>CCATCTTCAGCAAACCCTGATGAAGGCTACAAAGTAAGCGCAAG<br>TACCCACGTAAAGACGTTAGGTCAAGGTGTAGCCCATGAGGTGG<br>CAAGAAATGGGCTACATTTTCTACCCCAGAAAACTACGATAGCC<br>CTTATGAAACTTAAGGGTCGAAGGTGGATTTAGCAGTAAACTAA<br>GAGTAGAGTGCTTAGTTGAACAGGGCCCTGAAGCGCGTACACAC<br>CGCCCGTCACCCTCCTCAAGTATACTTCAAAGGACATTTAACTAA<br>AACCCCTACGCATTTATATAGAGGAG**A**CAAGTCGTAACATGGTA<br>AGTGTACTGGAAAGTGCACTTGGACGAAC

AATAGGTTTGGTCCTAGCCTTTCTATTAGCTCTTAGTAAGATTAC
ACATGCAAGCATCCCCGTTCCAGTGAGTTCACCCTCTAAATCACC
ACGATCAAAAGGAACAAGCATCAAGCACGCAGCAATGCAGCTC
AAAACGCTTAGCCTAGCCACACCCCCACGGGAAACAGCAGTGAT
TAACCTTTAGCAATAAACGAAAGTTTAACTAAGCTATACTAACC
CCAGGGTTGGTCAATTTCGTGCCAGCCACCGCGGTCACACGATT
AACCCAAGTCAATAGAAGCCGGCGTAAAGAGTGTTTTAGATCAC
CCCCTCCCCAATAAAGCTAAAACTCACCTGAGTTGTAAAAAACT
CCAGTTGACACAAAATAGACTACGAAAGTGGCTTTAACATATCT
GAACACACAATAGCTAAGACCCAAACTGGGATTAGATACCCCAC
TATGCTTAGCCCTAAACCTCAACAGTTAAATCAACAAAACTGCTC
1555G
GCCAGAACACTACGAGCCACAGCTTAAAACTCAAAGGACCTGGC
GGTGCTTCATATCCCTCTAGAGGAGCCTGTTCTGTAATCGATAAA
CCCCGATCAACCTCACCACCTCTTGCTCAGCCTATATACCGCCAT
CTTCAGCAAACCCTGATGAAGGCTACAAAGTAAGCGCAAGTACC
CACGTAAAGACGTTAGGTCAAGGTGTAGCCCATGAGGTGGCAAG
AAATGGGCTACATTTTCTACCCCAGAAAACTACGATAGCCCTTAT
GAAACTTAAGGGTCGAAGGTGGATTTAGCAGTAAACTAAGAGTA
GAGTGCTTAGTTGAACAGGGCCCTGAAGCGCGTACACACCGCCC
GTCACCCTCCTCAAGTATACTTCAAAGGACATTTAACTAAAACCC
CTACGCATTTATATAGAGGAG**G**CAAGTCGTAACATGGTAAGTGT
ACTGGAAAGTGCACTTGGACGAAC
