# Supplementary material for: Application of the TaqMan ARMS-PCR Approach for Genotyping Drug-Induced Hearing Loss Using Dried Blood Samples
Source: Curr Issues Mol Biol. 2024 May 29;46(6):0. doi: 10.3390/cimb46060326 (PMC13176787; doi:10.3390/cimb46060326)
Supplement: Supplementary file 1 [file cimb-46-00326-s001.zip › Table S3.pdf]

Table S3 Detection of DBS DNA samples by ARMS-PCR and Sanger sequencing

No.	Forward Sequencing	Reverse Sequencing	ARMS-PCR<br>for wild type detection	ARMS-PCR<br>for mutant type detection
1	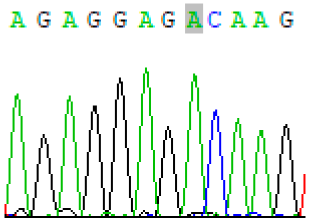	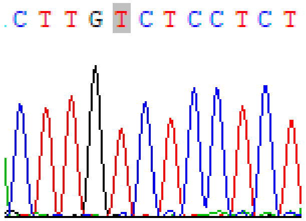	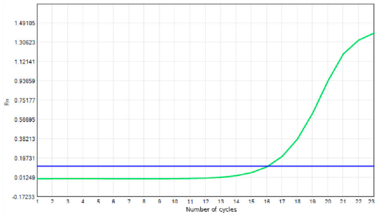	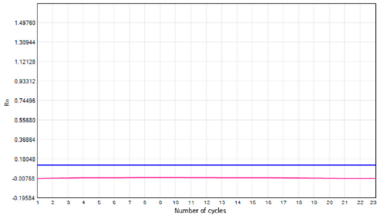
2	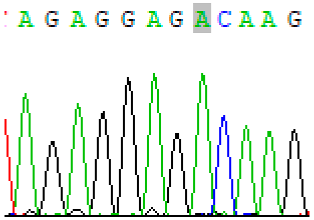	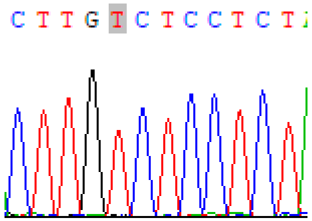	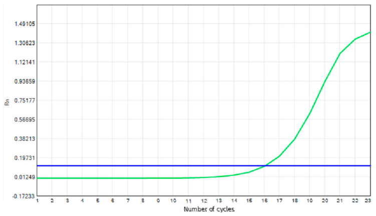	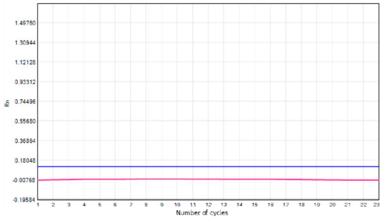
3	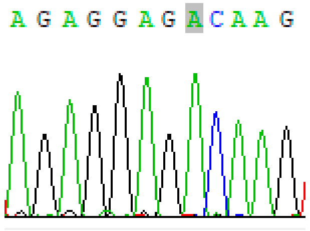	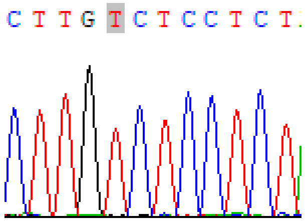	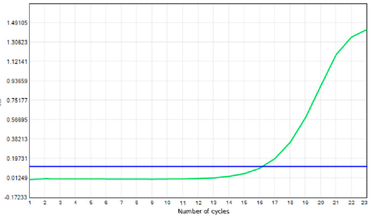	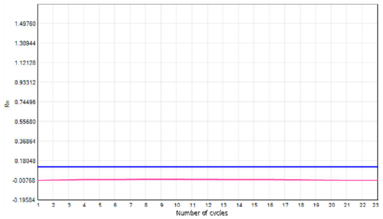
4	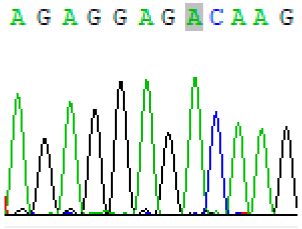	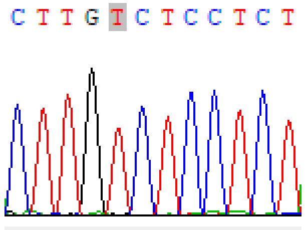	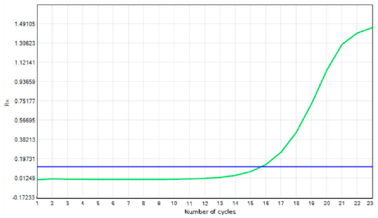	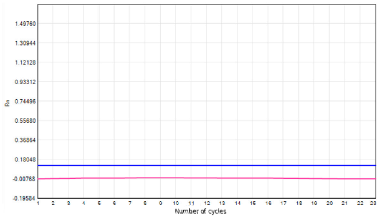

**5**	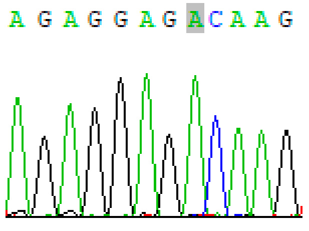A G A G G A G A C A A G	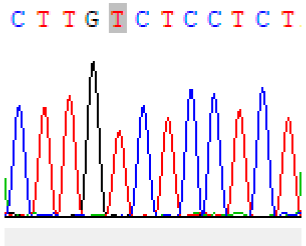C T T G T C T C C T C T	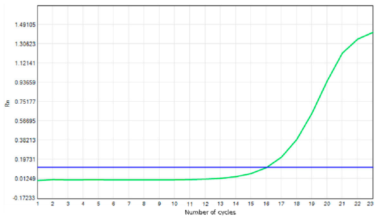	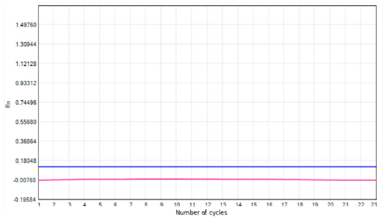
**6**	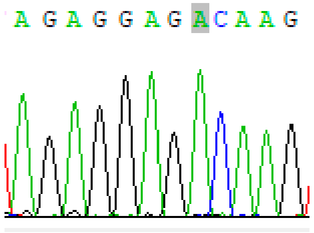A G A G G A G A C A A G	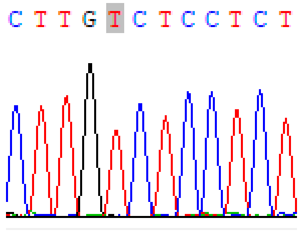C T T G T C T C C T C T	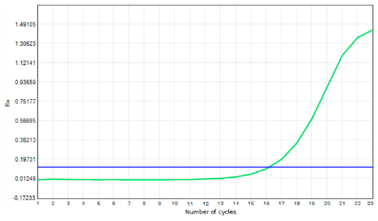	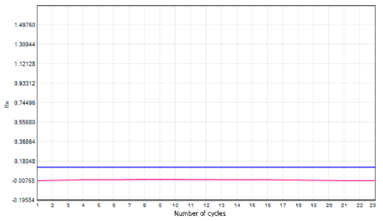
**7**	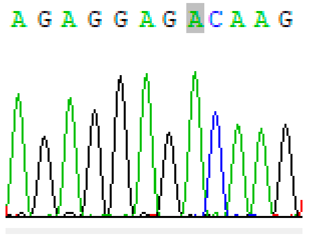A G A G G A G A C A A G	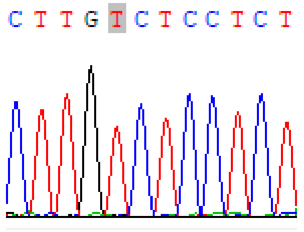C T T G T C T C C T C T	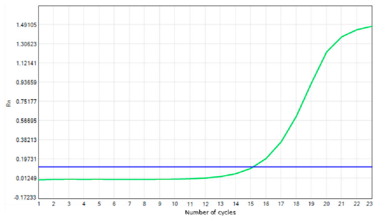	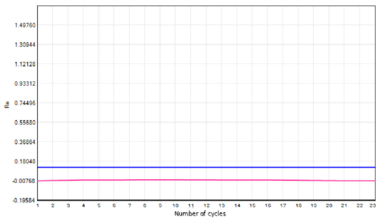
**8**	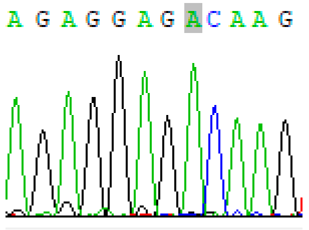A G A G G A G A C A A G	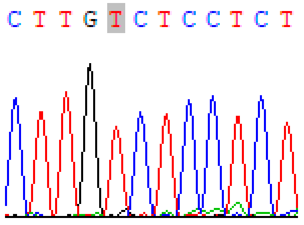C T T G T C T C C T C T	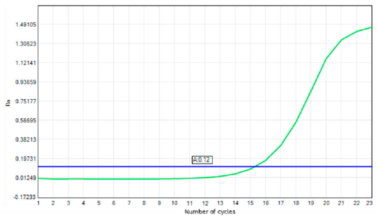	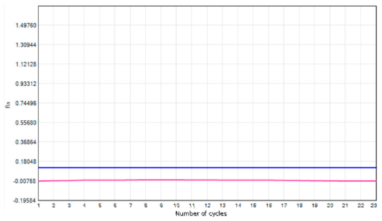
**9**	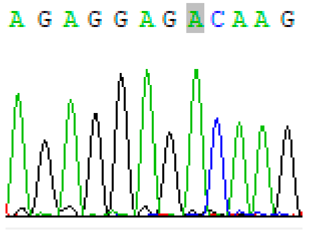A G A G G A G A C A A G	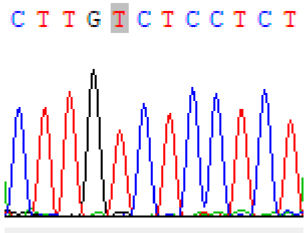C T T G T C T C C T C T	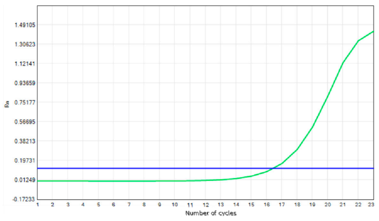	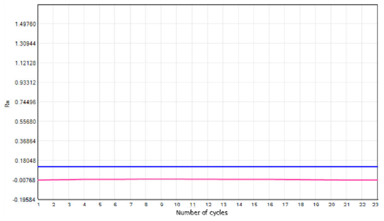

**10**	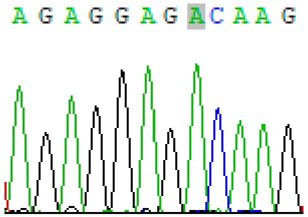A G A G G A G A C A A G	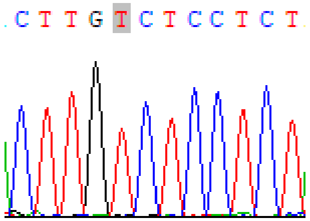C T T G T C T C C T C T	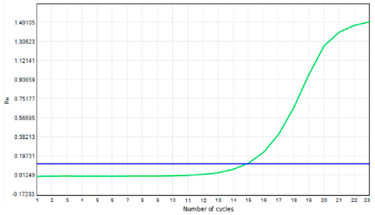Number of cycles	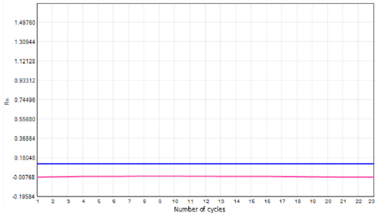Number of cycles
**11**	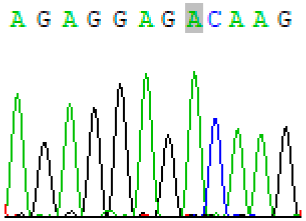A G A G G A G A C A A G	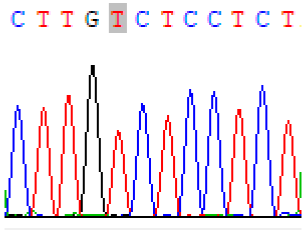C T T G T C T C C T C T	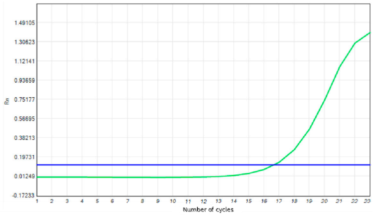Number of cycles	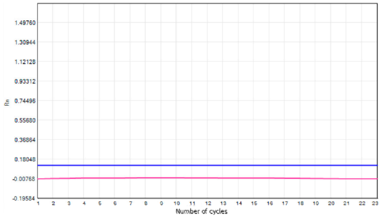Number of cycles
**12**	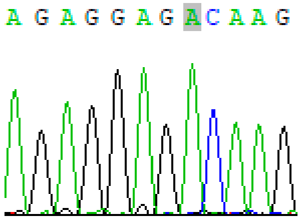A G A G G A G A C A A G	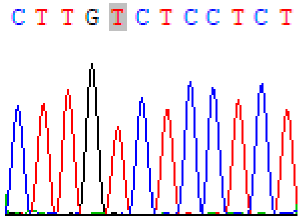C T T G T C T C C T C T	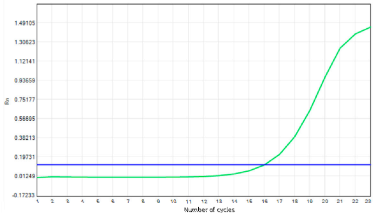Number of cycles	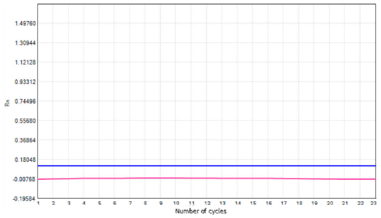Number of cycles
**13**	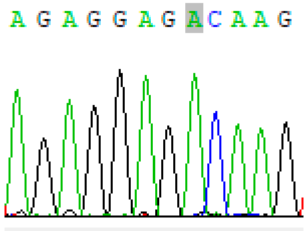A G A G G A G A C A A G	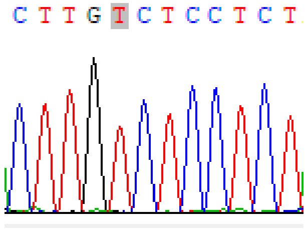C T T G T C T C C T C T	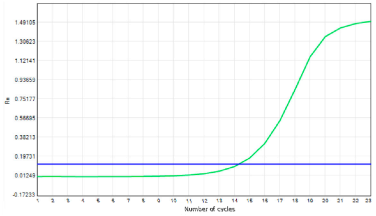Number of cycles	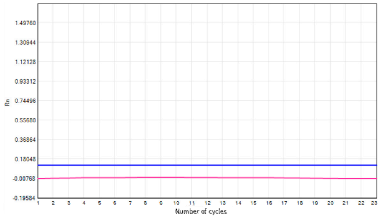Number of cycles
**14**	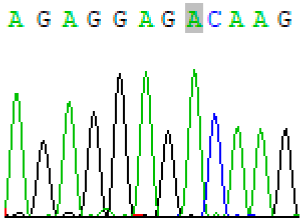A G A G G A G A C A A G	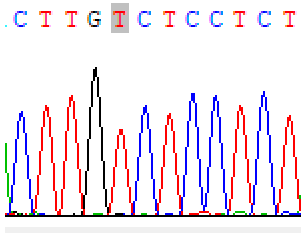C T T G T C T C C T C T	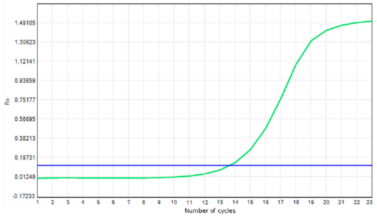Number of cycles	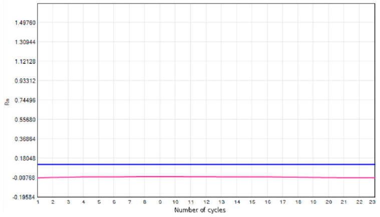Number of cycles

**15**	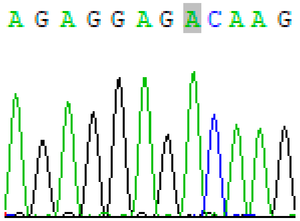A G A G G A G A C A A G	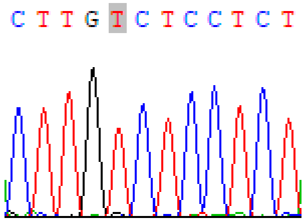C T T G T C T C C T C T	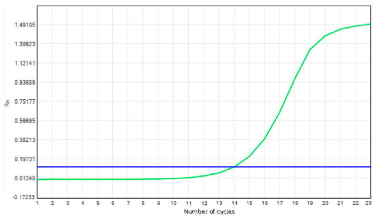Number of cycles	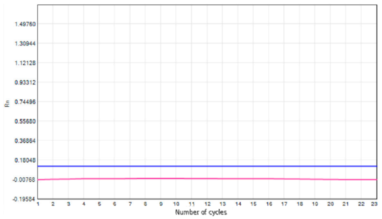Number of cycles
**16**	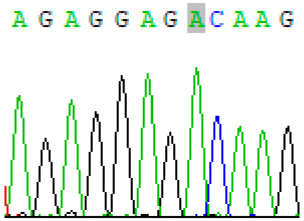A G A G G A G A C A A G	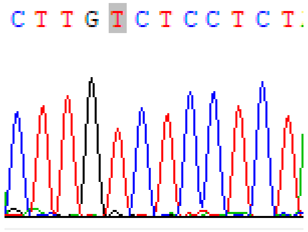C T T G T C T C C T C T	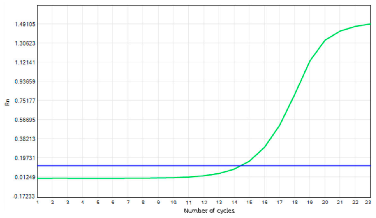Number of cycles	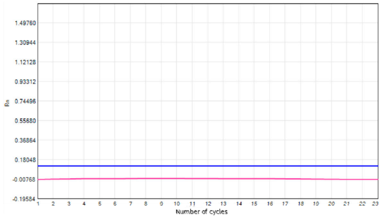Number of cycles
**17**	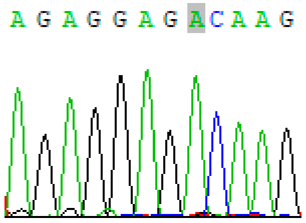A G A G G A G A C A A G	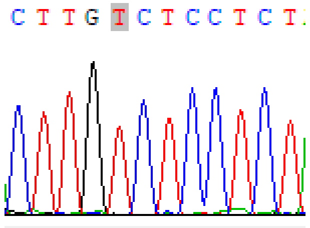C T T G T C T C C T C T	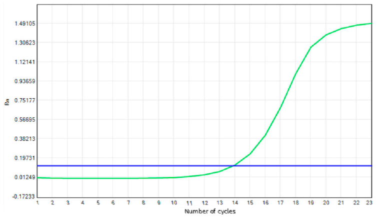Number of cycles	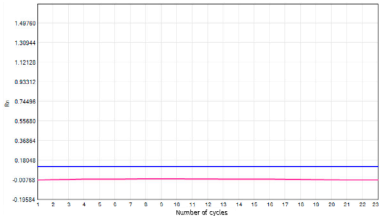Number of cycles
**18**	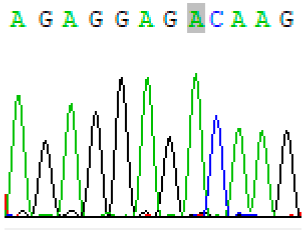A G A G G A G A C A A G	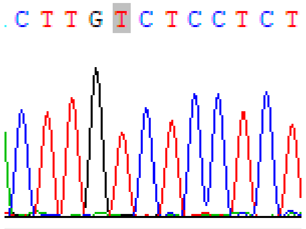C T T G T C T C C T C T	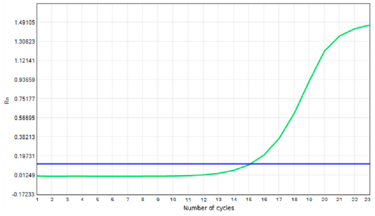Number of cycles	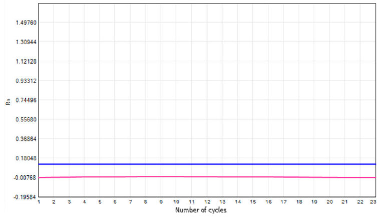Number of cycles
**19**	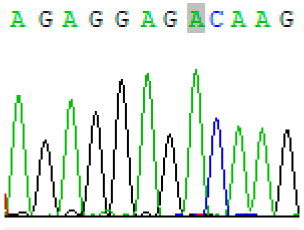A G A G G A G A C A A G	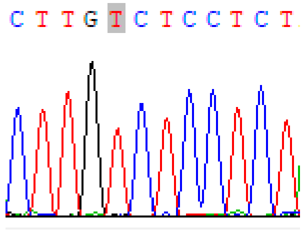C T T G T C T C C T C T	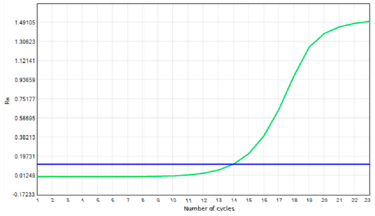Number of cycles	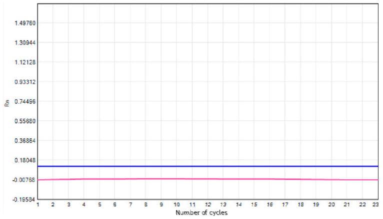Number of cycles

**20**	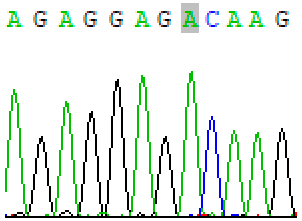 A G A G G A G A C A A G	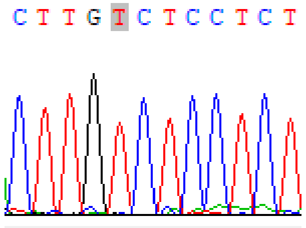 C T T G T C T C C T C T	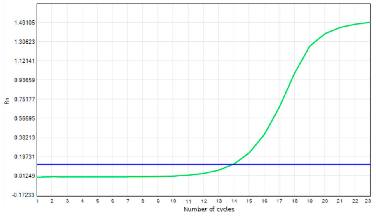	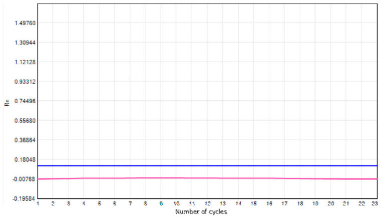
**21**	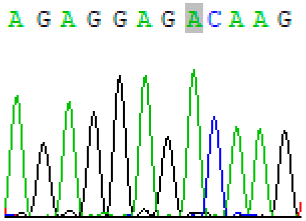 A G A G G A G A C A A G	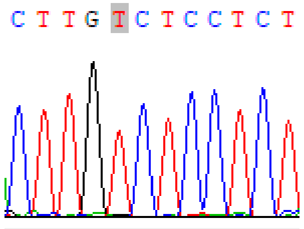 C T T G T C T C C T C T	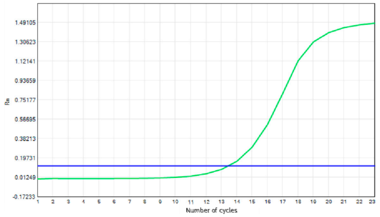	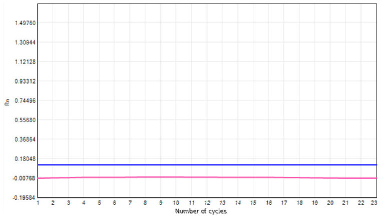
**22**	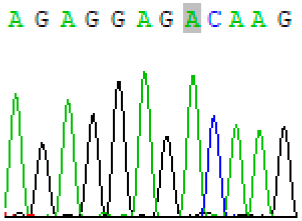 A G A G G A G A C A A G	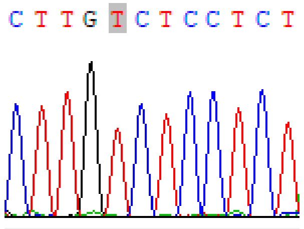 C T T G T C T C C T C T	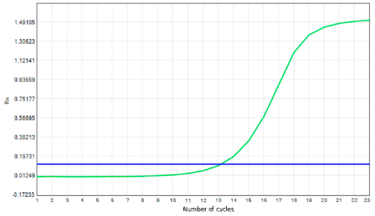	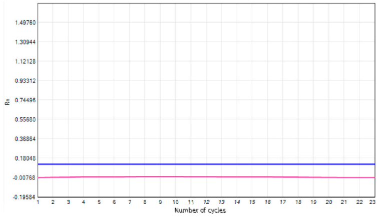
**23**	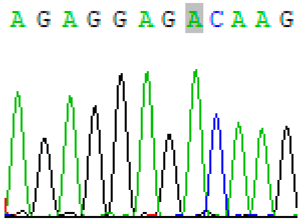 A G A G G A G A C A A G	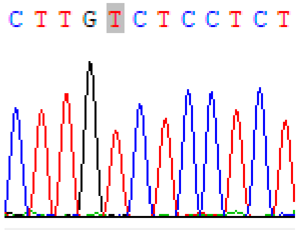 C T T G T C T C C T C T	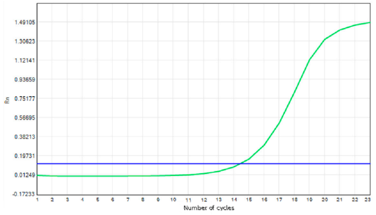	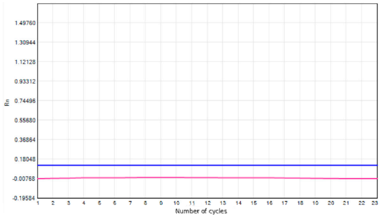
**24**	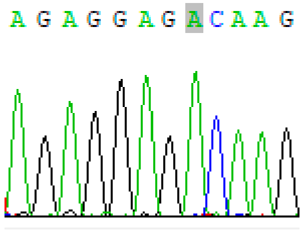 A G A G G A G A C A A G	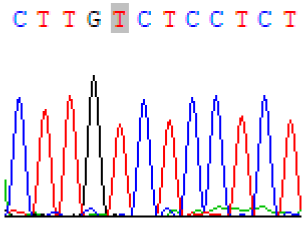 C T T G T C T C C T C T	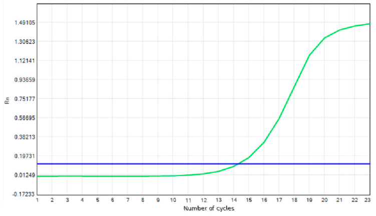	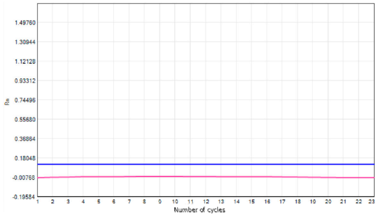

**25**	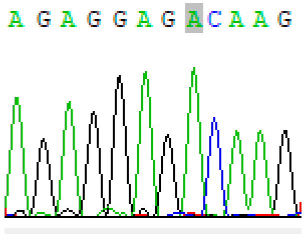 A G A G G A G A C A A G	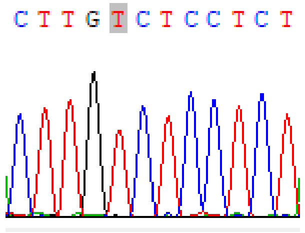 C T T G T C T C C T C T	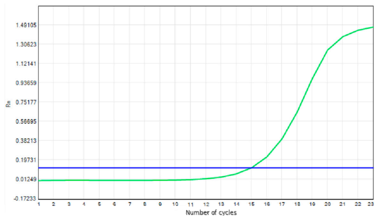 Number of cycles	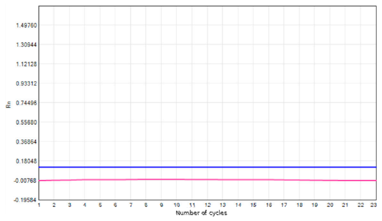 Number of cycles
**26**	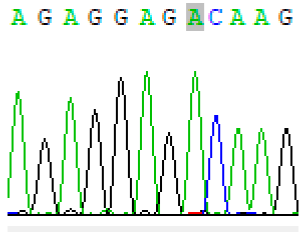 A G A G G A G A C A A G	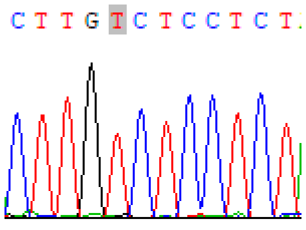 C T T G T C T C C T C T	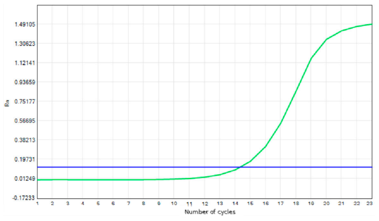 Number of cycles	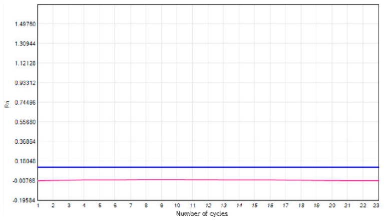 Number of cycles
**27**	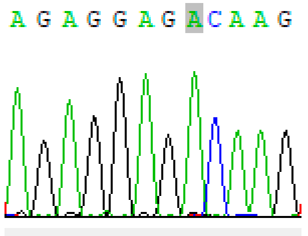 A G A G G A G A C A A G	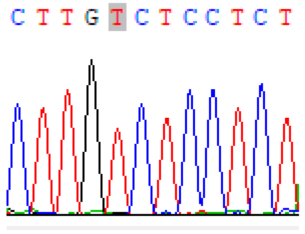 C T T G T C T C C T C T	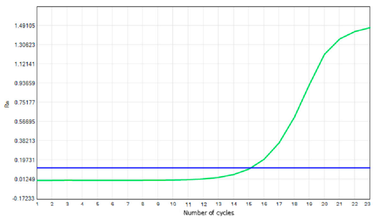 Number of cycles	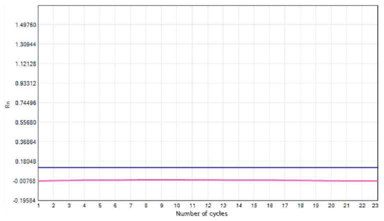 Number of cycles
**28**	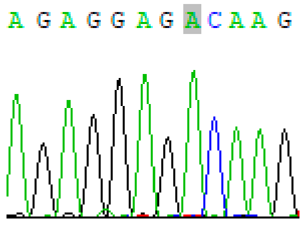 A G A G G A G A C A A G	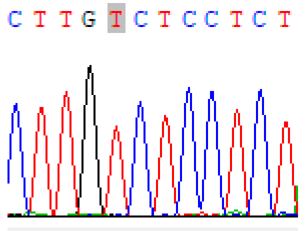 C T T G T C T C C T C T	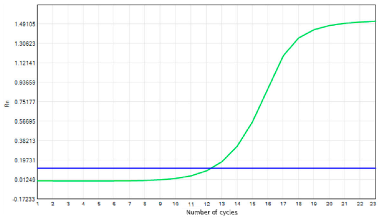 Number of cycles	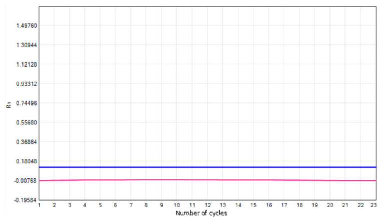 Number of cycles
**29**	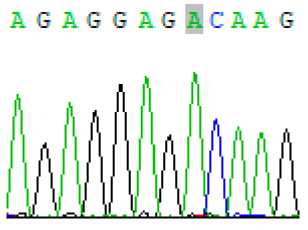 A G A G G A G A C A A G	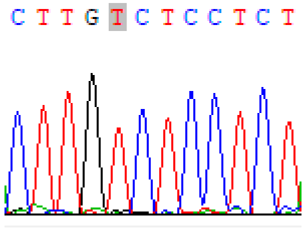 C T T G T C T C C T C T	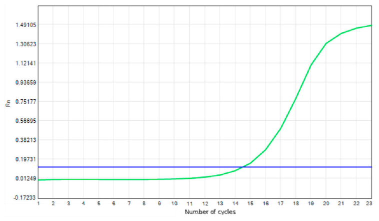 Number of cycles	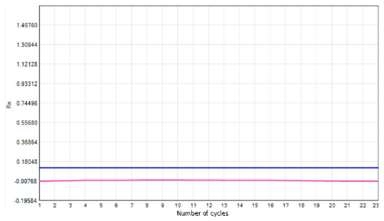 Number of cycles

**30**	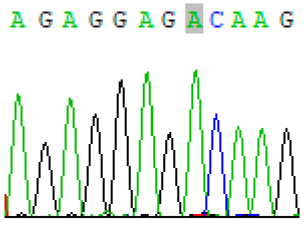A G A G G A G A C A A G	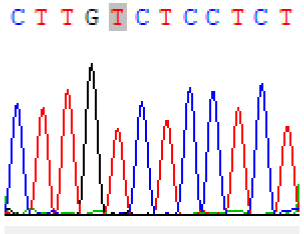C T T G T C T C C T C T	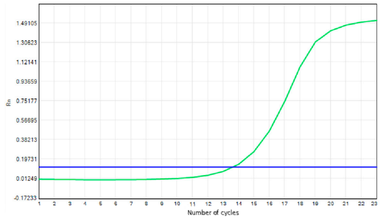Number of cycles	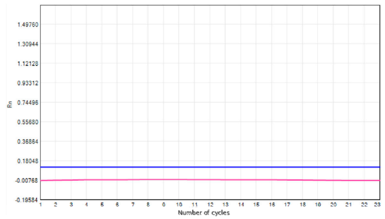Number of cycles
**31**	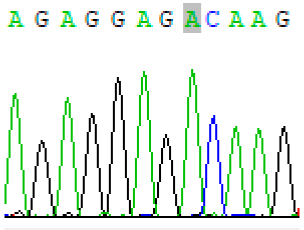A G A G G A G A C A A G	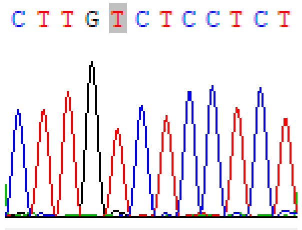C T T G T C T C C T C T	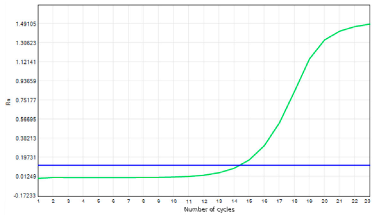Number of cycles	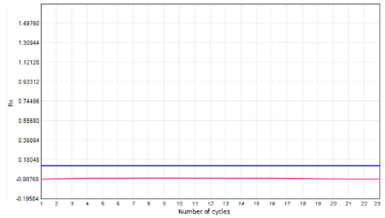Number of cycles
**32**	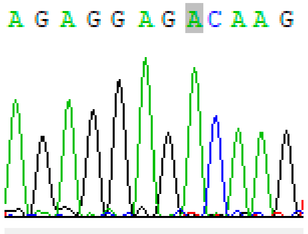A G A G G A G A C A A G	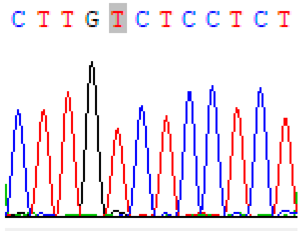C T T G T C T C C T C T	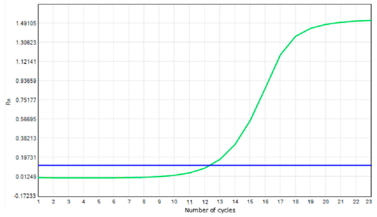Number of cycles	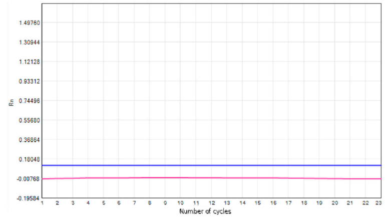Number of cycles
**33**	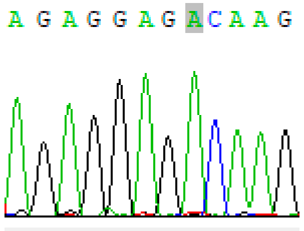A G A G G A G A C A A G	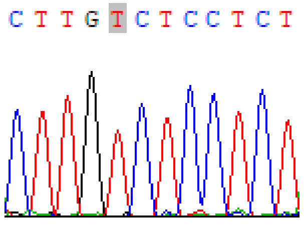C T T G T C T C C T C T	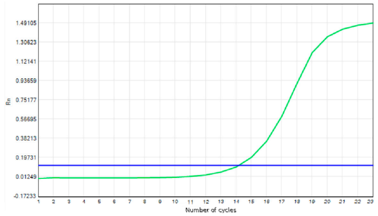Number of cycles	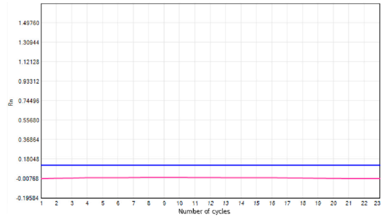Number of cycles
**34**	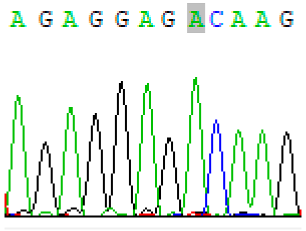A G A G G A G A C A A G	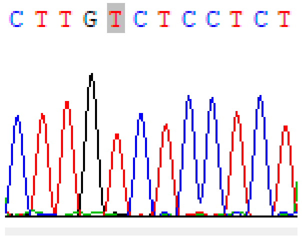C T T G T C T C C T C T	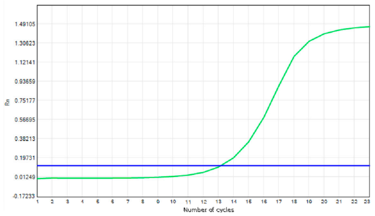Number of cycles	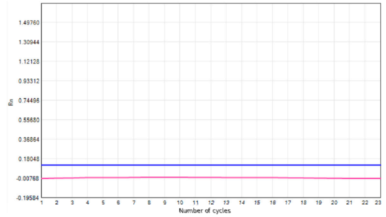Number of cycles

**35**	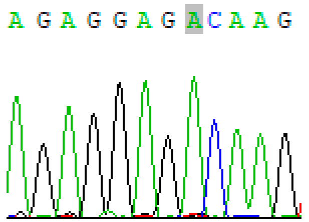A G A G G A G A C A A G	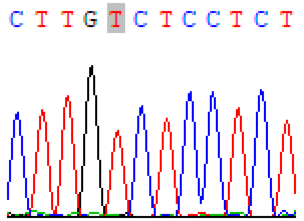C T T G T C T C C T C T	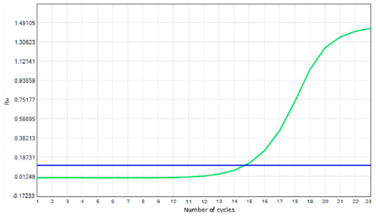Number of cycles	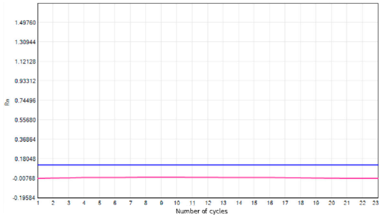Number of cycles
**36**	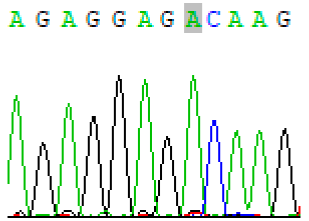A G A G G A G A C A A G	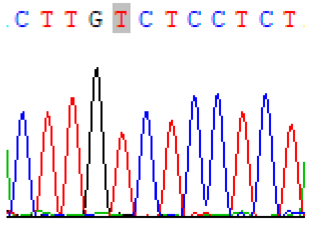C T T G T C T C C T C T	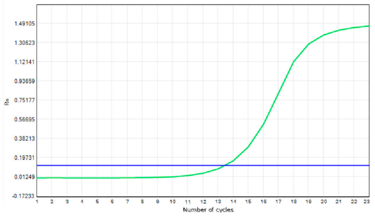Number of cycles	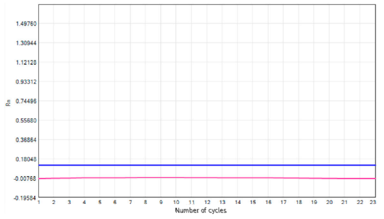Number of cycles
**37**	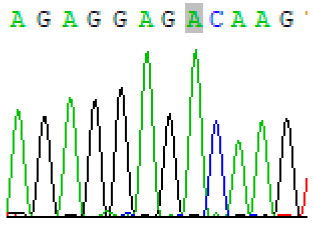A G A G G A G A C A A G	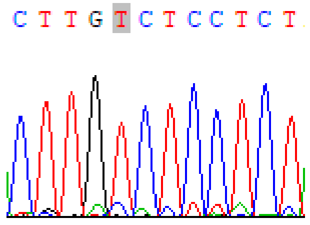C T T G T C T C C T C T	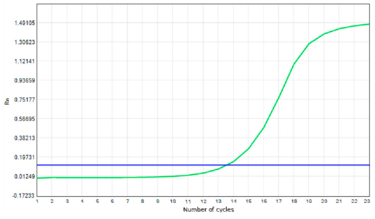Number of cycles	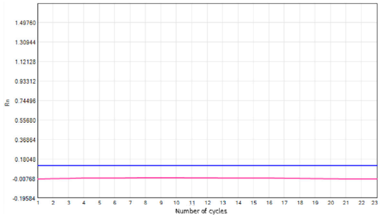Number of cycles
**38**	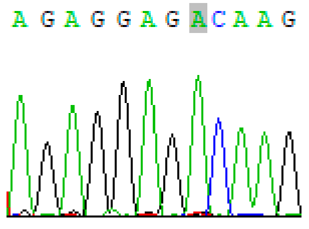A G A G G A G A C A A G	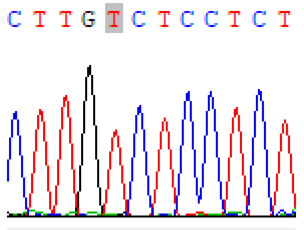C T T G T C T C C T C T	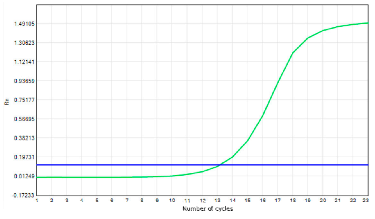Number of cycles	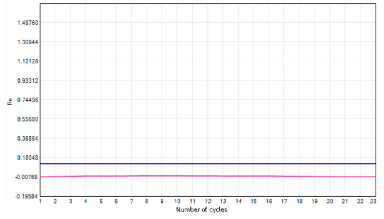Number of cycles
**39**	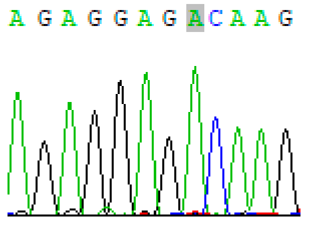A G A G G A G A C A A G	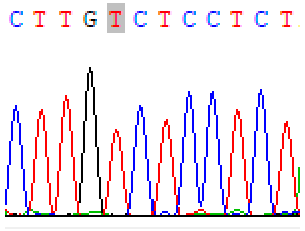C T T G T C T C C T C T	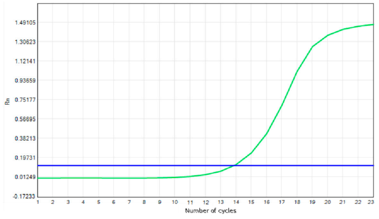Number of cycles	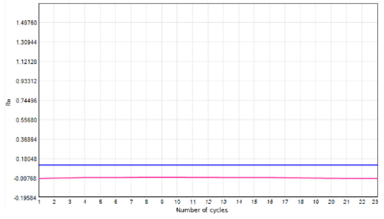Number of cycles

**40**	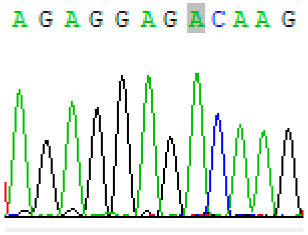 A G A G G A G A C A A G	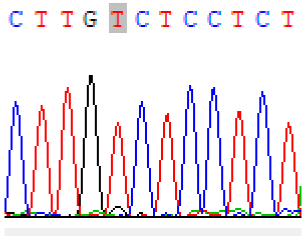 C T T G T C T C C T C T	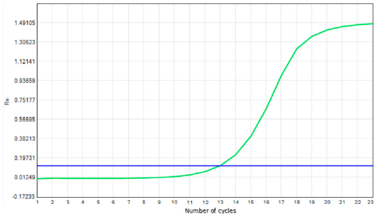 Number of cycles	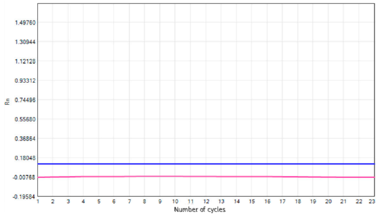 Number of cycles
**41**	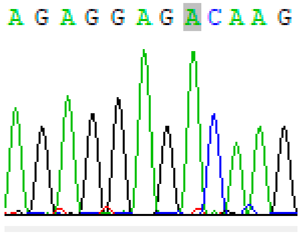 A G A G G A G A C A A G	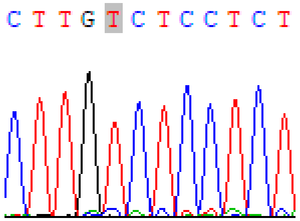 C T T G T C T C C T C T	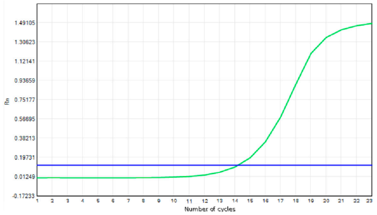 Number of cycles	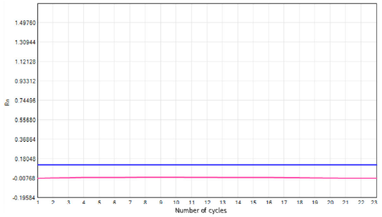 Number of cycles
**42**	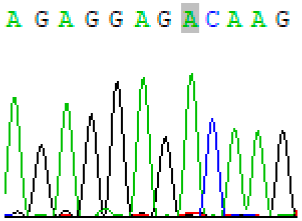 A G A G G A G A C A A G	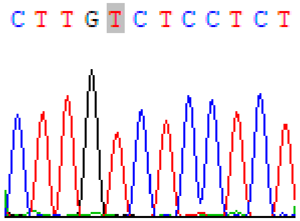 C T T G T C T C C T C T	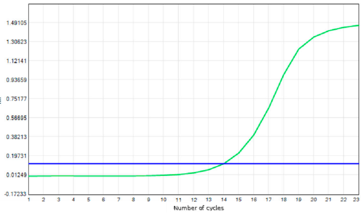 Number of cycles	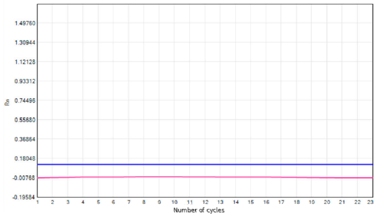 Number of cycles
**43**	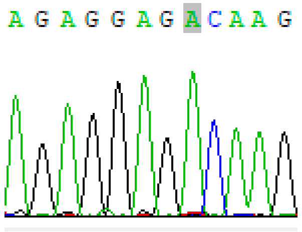 A G A G G A G A C A A G	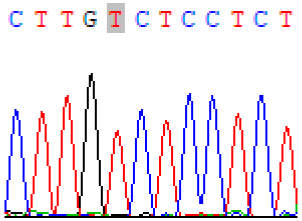 C T T G T C T C C T C T	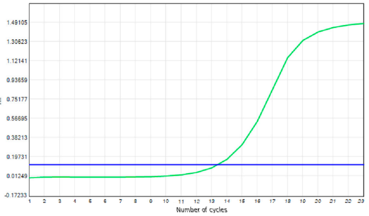 Number of cycles	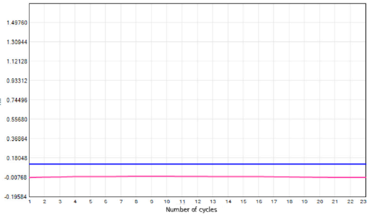 Number of cycles
**44**	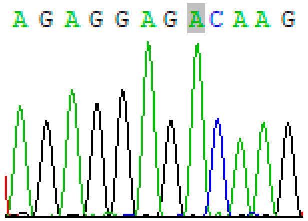 A G A G G A G A C A A G	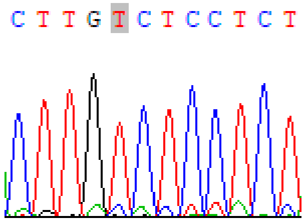 C T T G T C T C C T C T	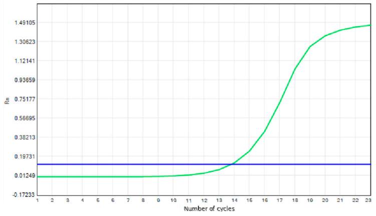 Number of cycles	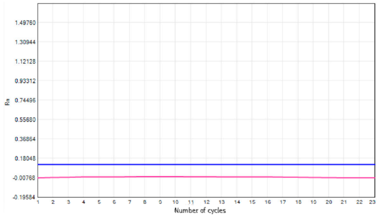 Number of cycles

**45**	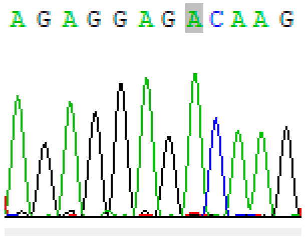A G A G G A G A C A A G	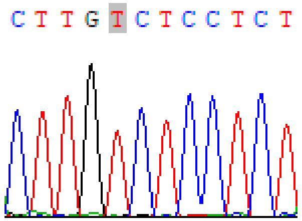C T T G T C T C C T C T	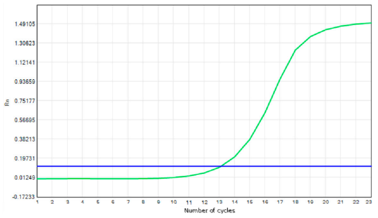Number of cycles	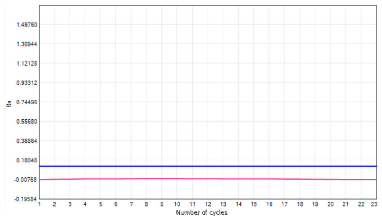Number of cycles
**46**	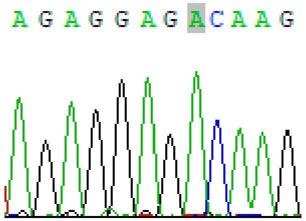A G A G G A G A C A A G	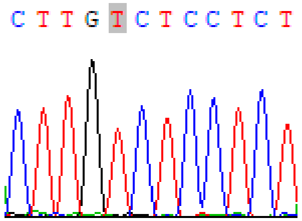C T T G T C T C C T C T	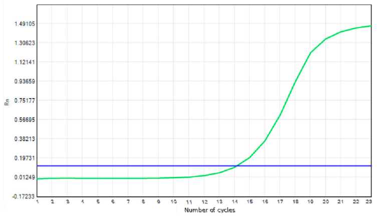Number of cycles	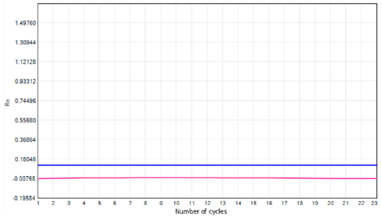Number of cycles
**47**	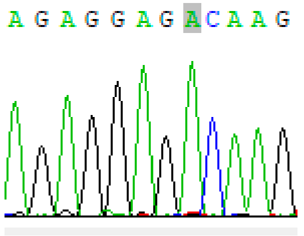A G A G G A G A C A A G	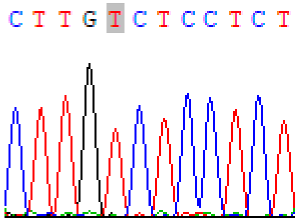C T T G T C T C C T C T	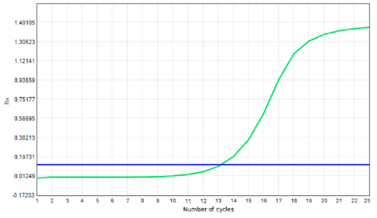Number of cycles	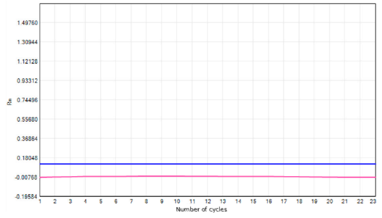Number of cycles
**48**	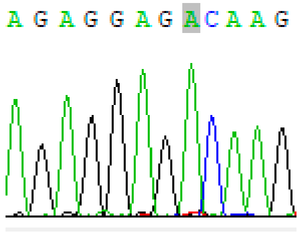A G A G G A G A C A A G	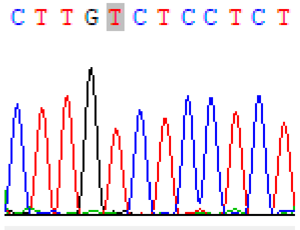C T T G T C T C C T C T	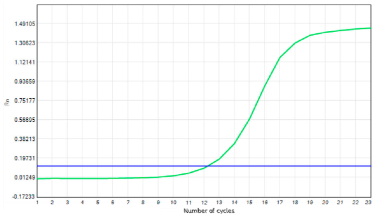Number of cycles	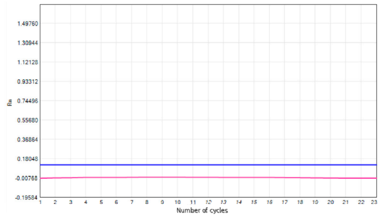Number of cycles
**49**	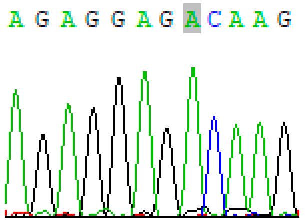A G A G G A G A C A A G	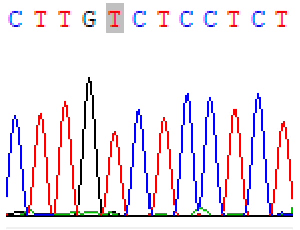C T T G T C T C C T C T	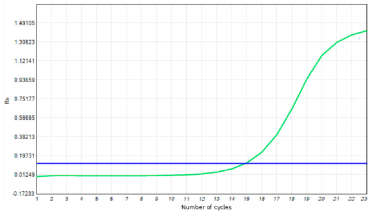Number of cycles	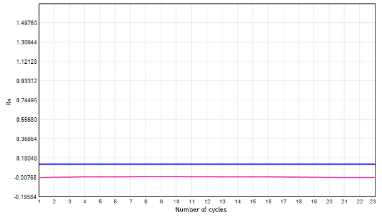Number of cycles

**50**	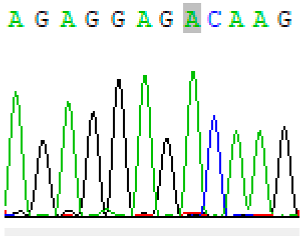 A G A G G A G A C A A G	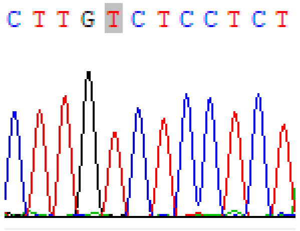 C T T G T C T C C T C T	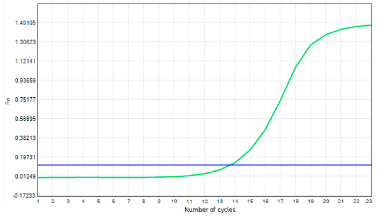	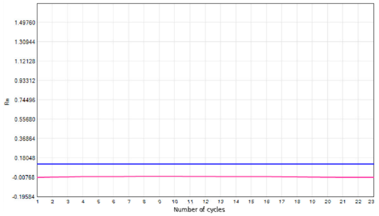
**51**	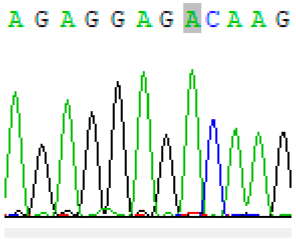 A G A G G A G A C A A G	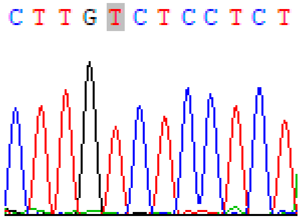 C T T G T C T C C T C T	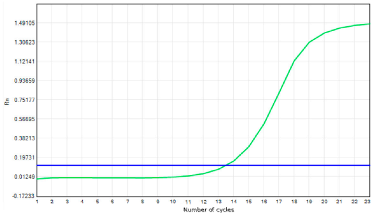	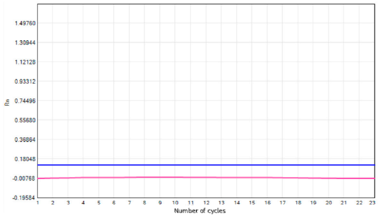
**52**	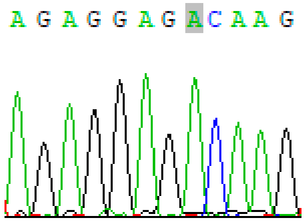 A G A G G A G A C A A G	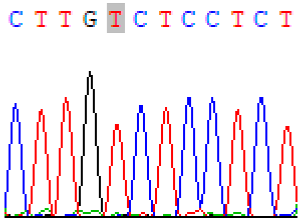 C T T G T C T C C T C T	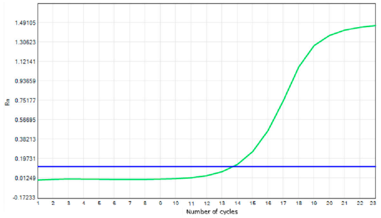	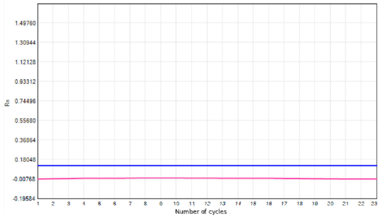
**53**	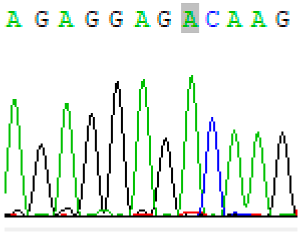 A G A G G A G A C A A G	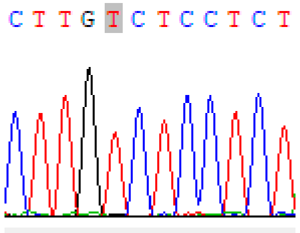 C T T G T C T C C T C T	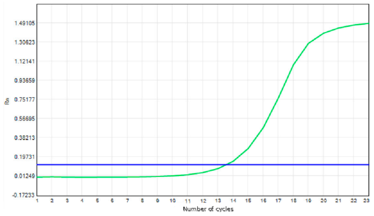	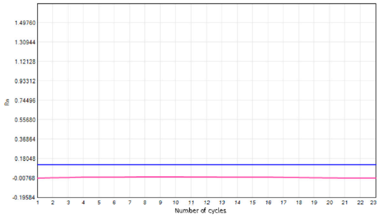
**54**	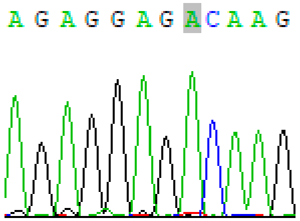 A G A G G A G A C A A G	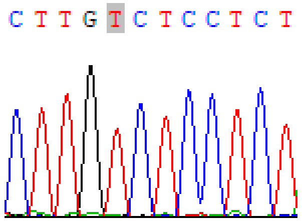 C T T G T C T C C T C T	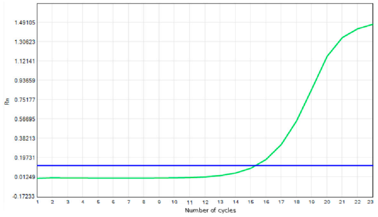	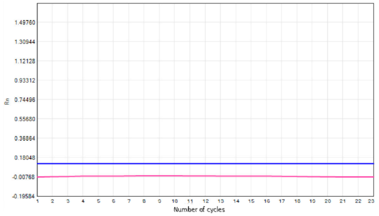

**55**	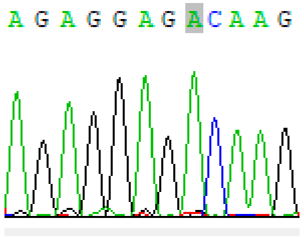A G A G G A G A C A A G	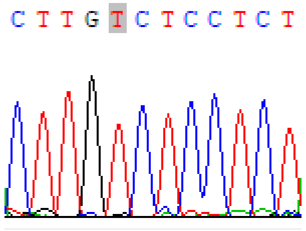C T T G T C T C C T C T	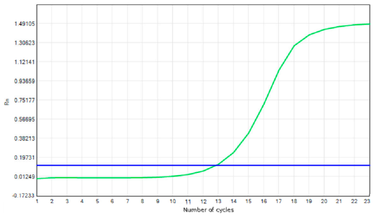Number of cycles	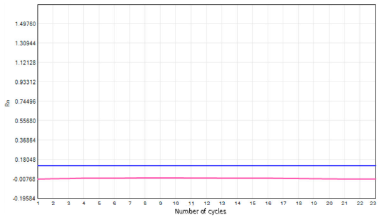Number of cycles
**56**	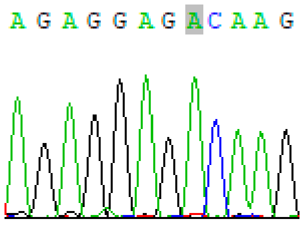A G A G G A G A C A A G	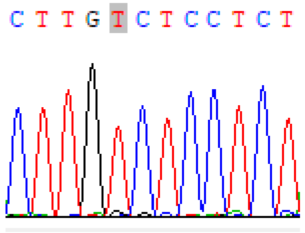C T T G T C T C C T C T	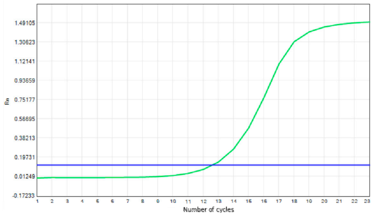Number of cycles	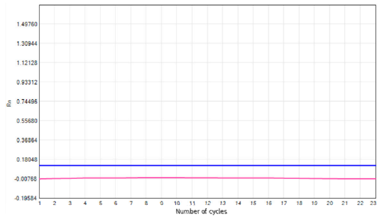Number of cycles
**57**	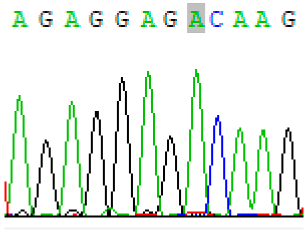A G A G G A G A C A A G	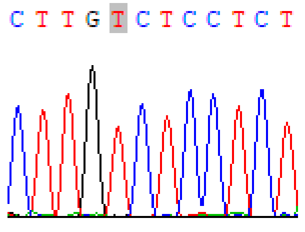C T T G T C T C C T C T	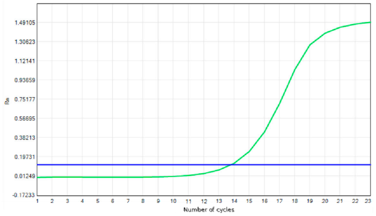Number of cycles	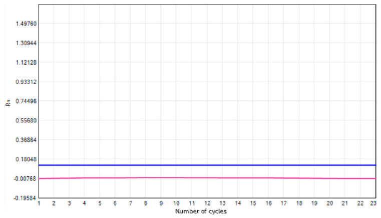Number of cycles
**58**	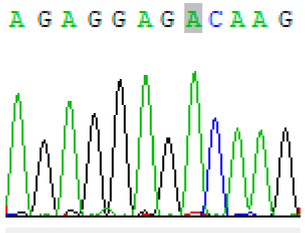A G A G G A G A C A A G	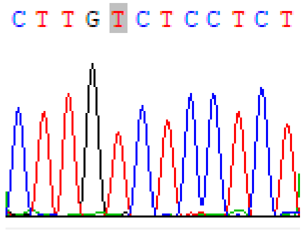C T T G T C T C C T C T	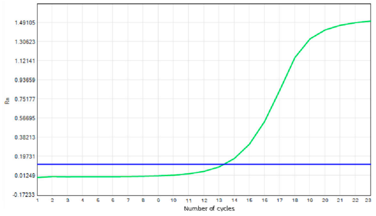Number of cycles	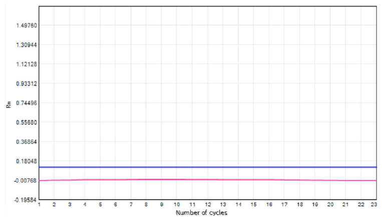Number of cycles
**59**	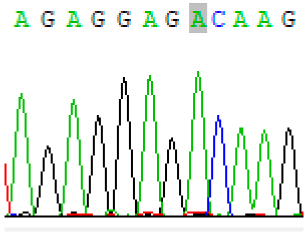A G A G G A G A C A A G	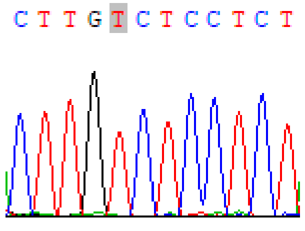C T T G T C T C C T C T	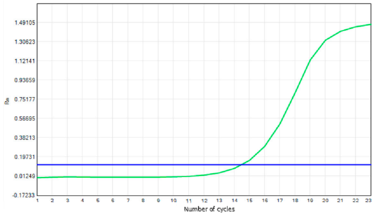Number of cycles	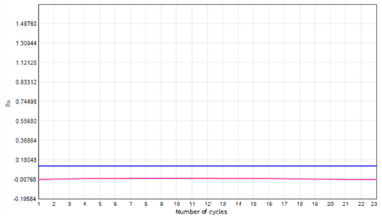Number of cycles

**60**	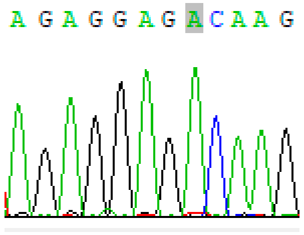 A G A G G A G A C A A G	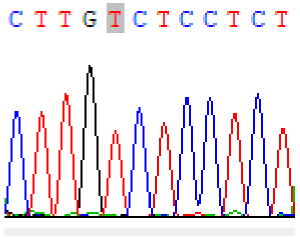 C T T G T C T C C T C T	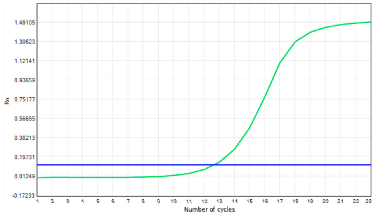 Number of cycles	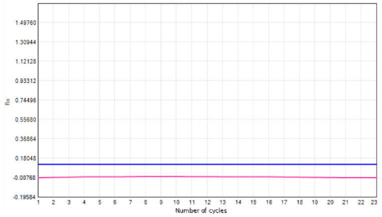 Number of cycles
**61**	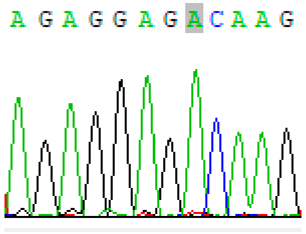 A G A G G A G A C A A G	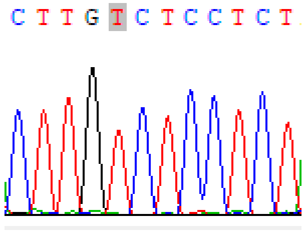 C T T G T C T C C T C T	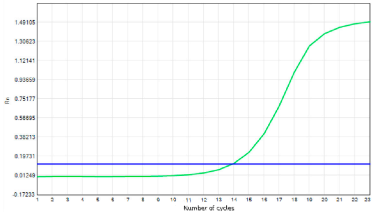 Number of cycles	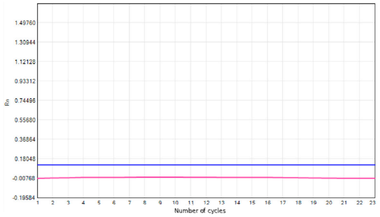 Number of cycles
**62**	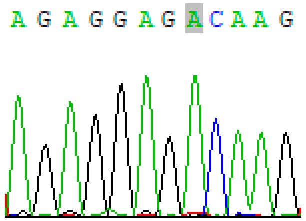 A G A G G A G A C A A G	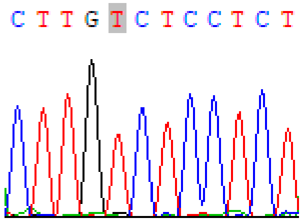 C T T G T C T C C T C T	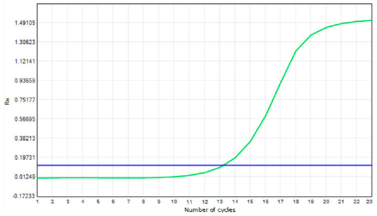 Number of cycles	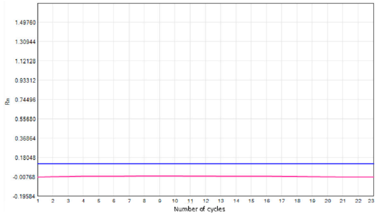 Number of cycles
**63**	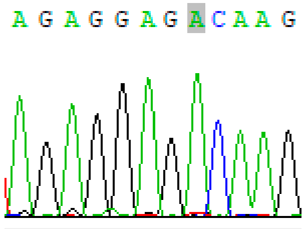 A G A G G A G A C A A G	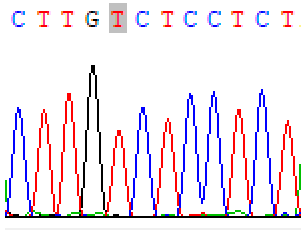 C T T G T C T C C T C T	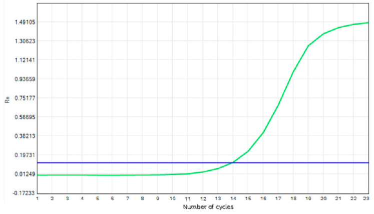 Number of cycles	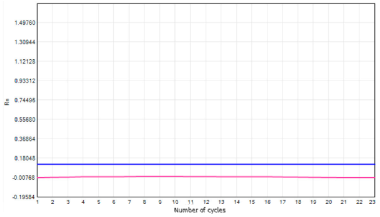 Number of cycles
**64**	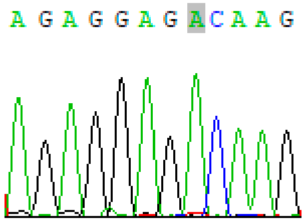 A G A G G A G A C A A G	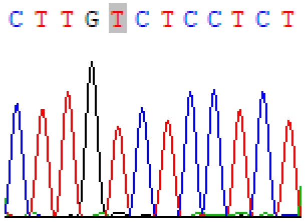 C T T G T C T C C T C T	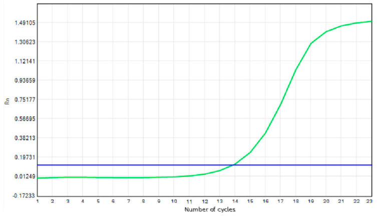 Number of cycles	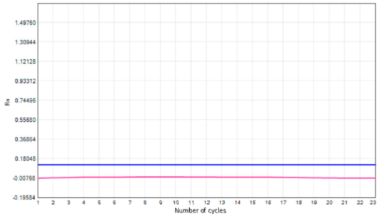 Number of cycles

**65**	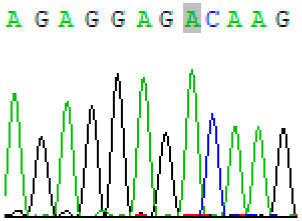 A G A G G A G A C A A G	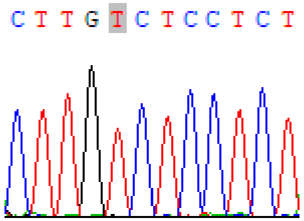 C T T G T C T C C T C T	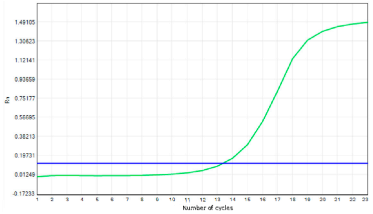 Number of cycles	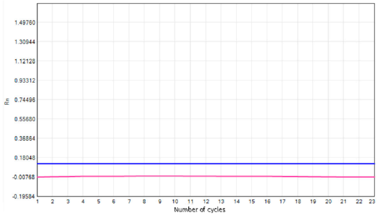 Number of cycles
**66**	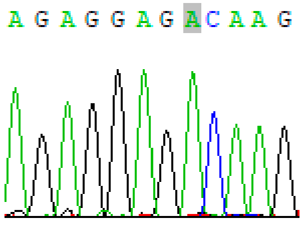 A G A G G A G A C A A G	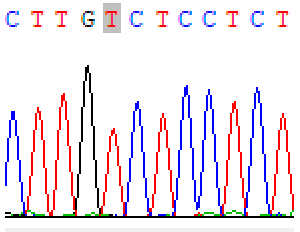 C T T G T C T C C T C T	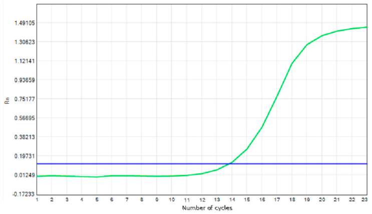 Number of cycles	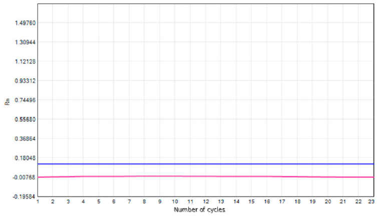 Number of cycles
**67**	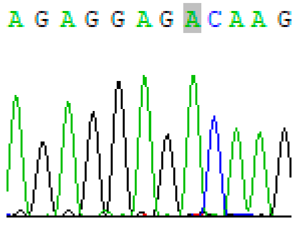 A G A G G A G A C A A G	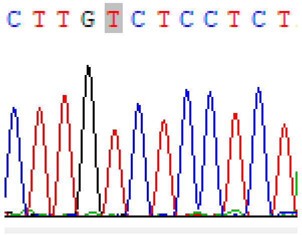 C T T G T C T C C T C T	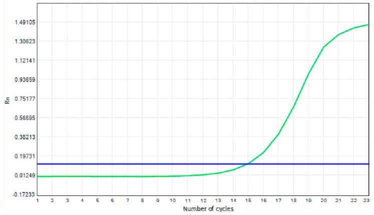 Number of cycles	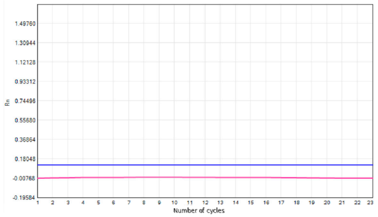 Number of cycles
**68**	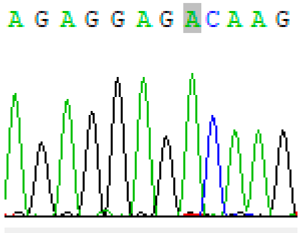 A G A G G A G A C A A G	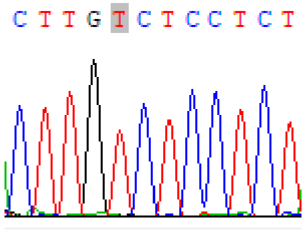 C T T G T C T C C T C T	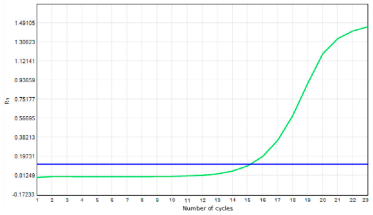 Number of cycles	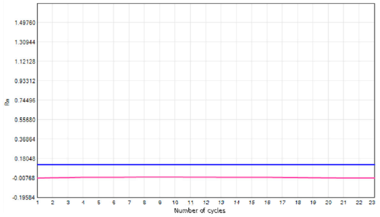 Number of cycles
**69**	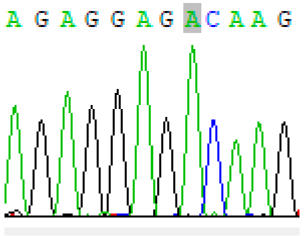 A G A G G A G A C A A G	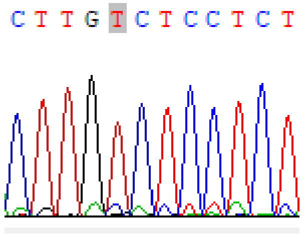 C T T G T C T C C T C T	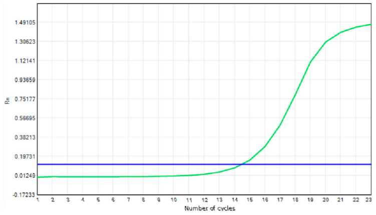 Number of cycles	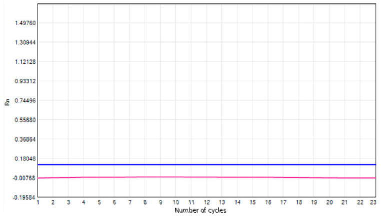 Number of cycles

**70**	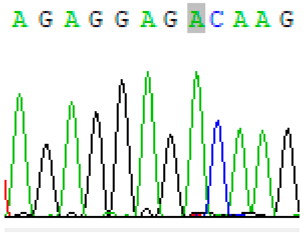A G A G G A G A C A A G	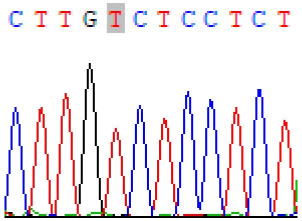C T T G T C T C C T C T	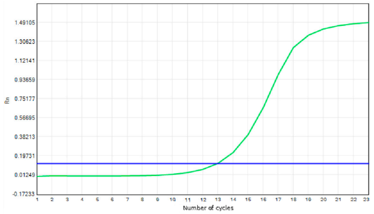	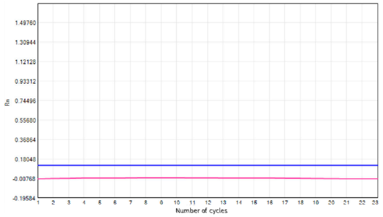
**71**	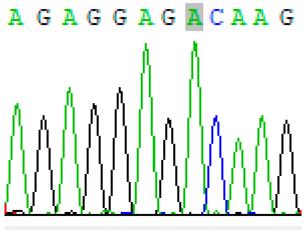A G A G G A G A C A A G	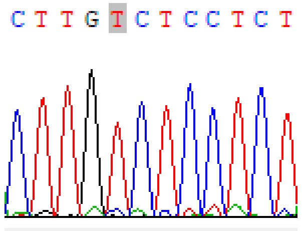C T T G T C T C C T C T	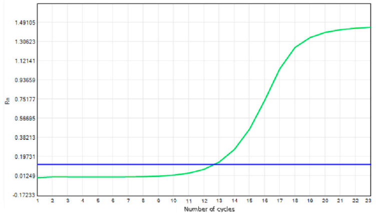	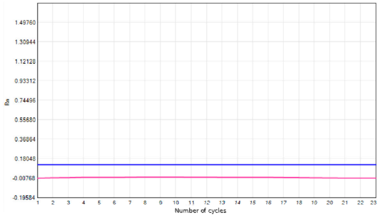
**72**	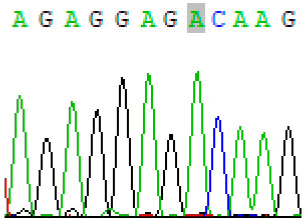A G A G G A G A C A A G	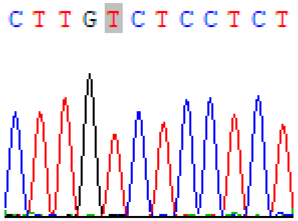C T T G T C T C C T C T	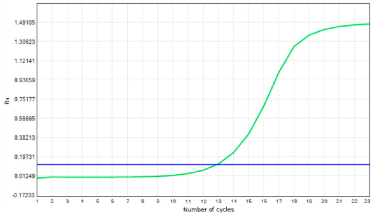	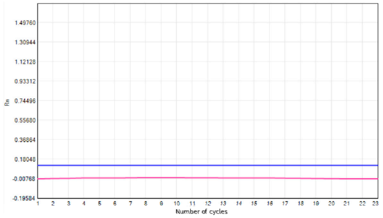
**73**	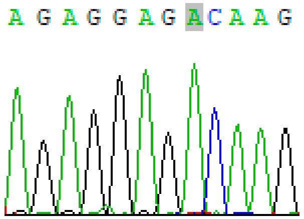A G A G G A G A C A A G	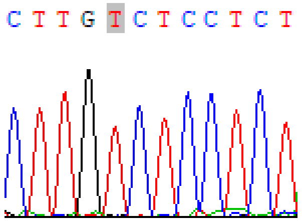C T T G T C T C C T C T	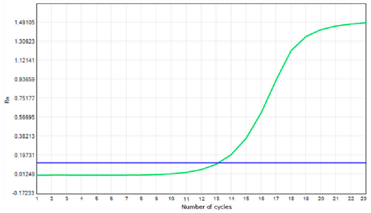	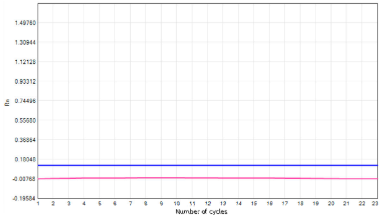
**74**	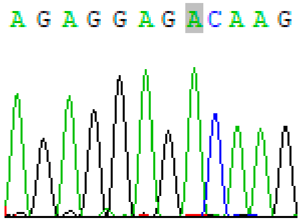A G A G G A G A C A A G	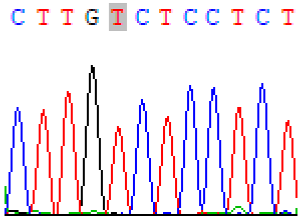C T T G T C T C C T C T	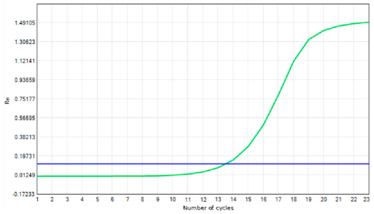	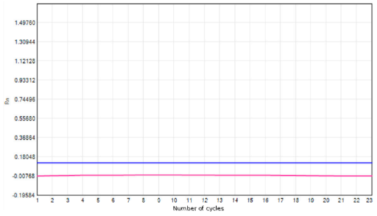

**75**	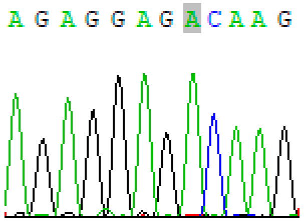 A G A G G A G A C A A G	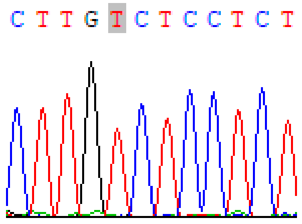 C T T G T C T C C T C T	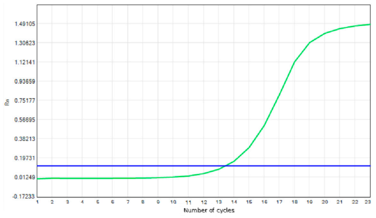	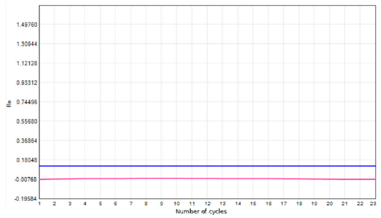
**76**	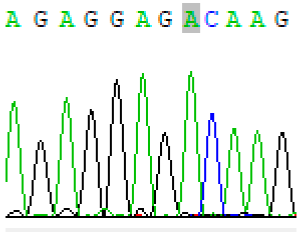 A G A G G A G A C A A G	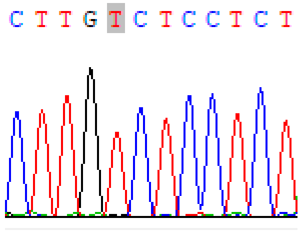 C T T G T C T C C T C T	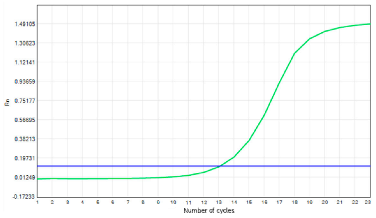	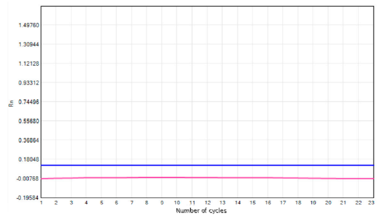
**77**	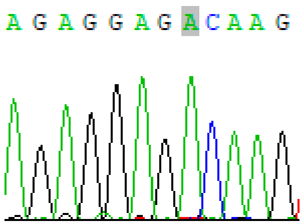 A G A G G A G A C A A G	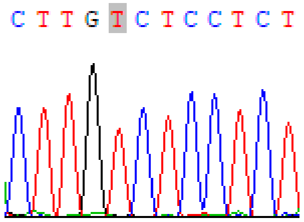 C T T G T C T C C T C T	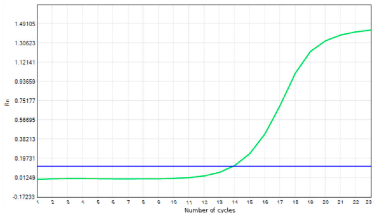	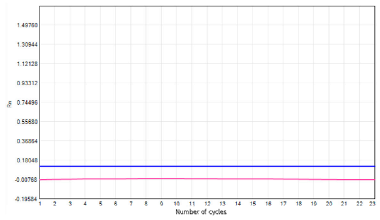
**78**	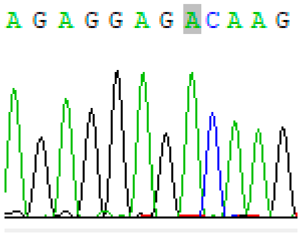 A G A G G A G A C A A G	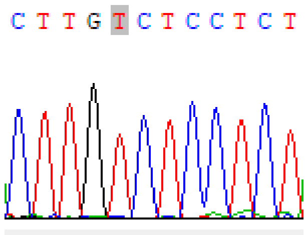 C T T G T C T C C T C T	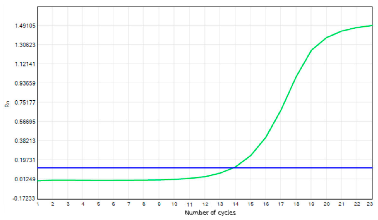	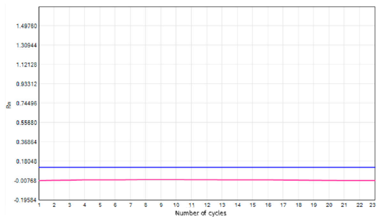
**79**	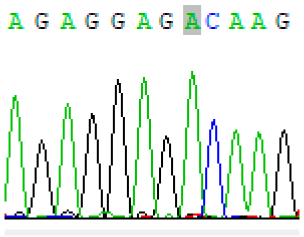 A G A G G A G A C A A G	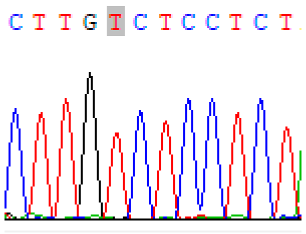 C T T G T C T C C T C T	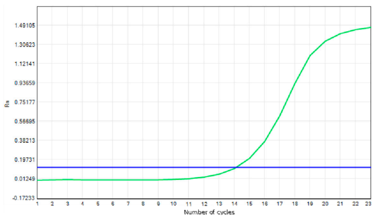	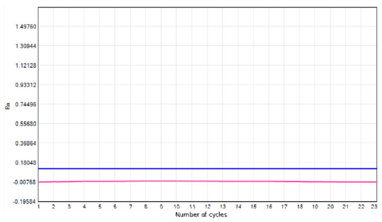

**80**	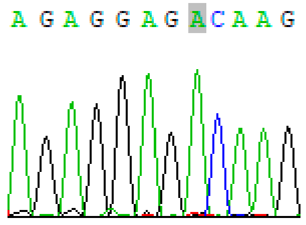 A G A G G A G A C A A G	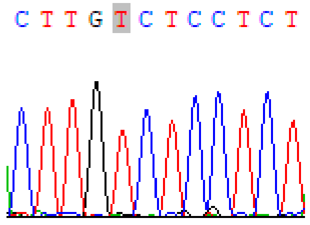 C T T G T C T C C T C T	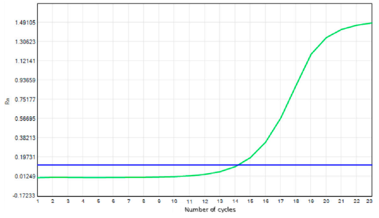	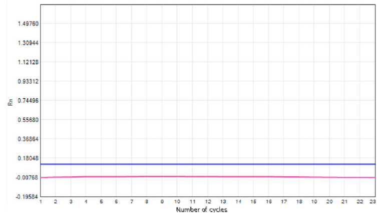
**81**	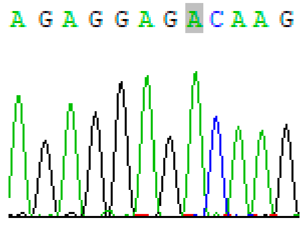 A G A G G A G A C A A G	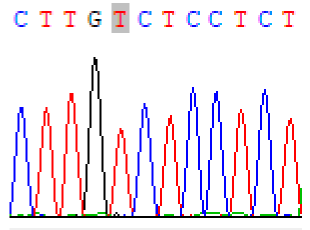 C T T G T C T C C T C T	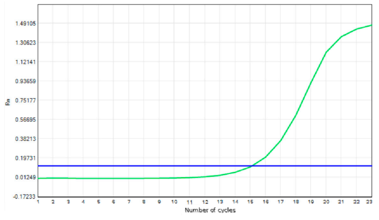	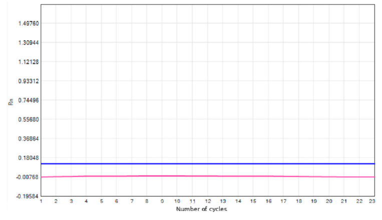
**82**	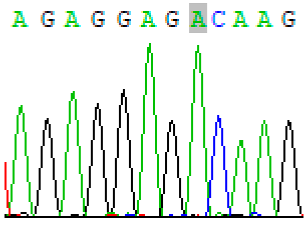 A G A G G A G A C A A G	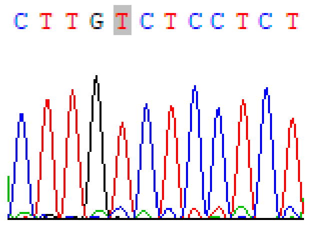 C T T G T C T C C T C T	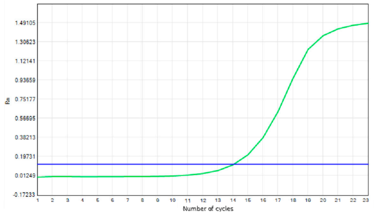	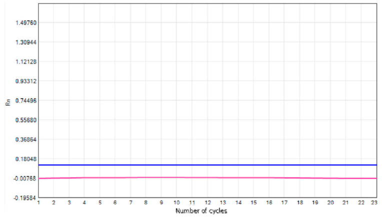
**83**	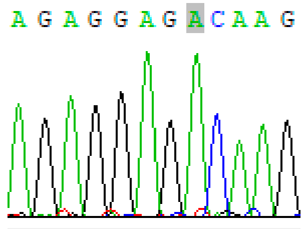 A G A G G A G A C A A G	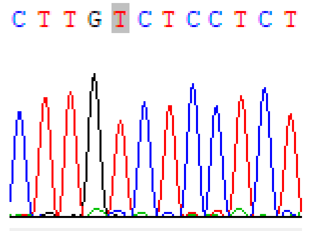 C T T G T C T C C T C T	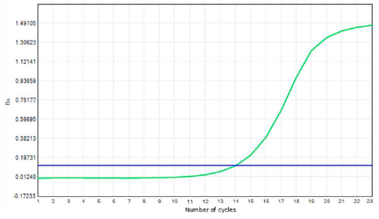	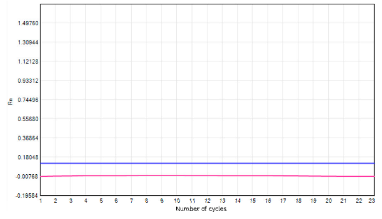
**84**	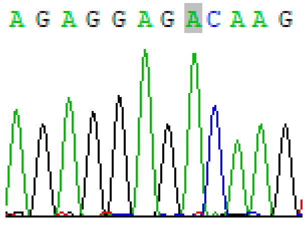 A G A G G A G A C A A G	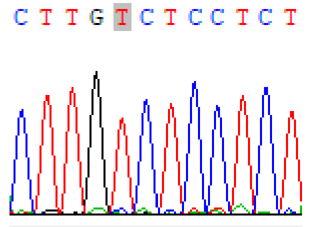 C T T G T C T C C T C T	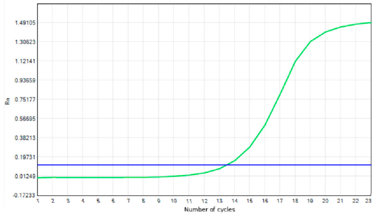	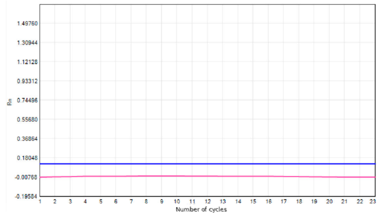

**85**	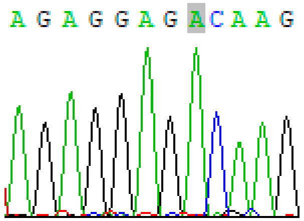 A G A G G A G A C A A G	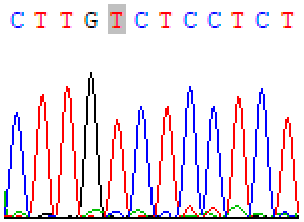 C T T G T C T C C T C T	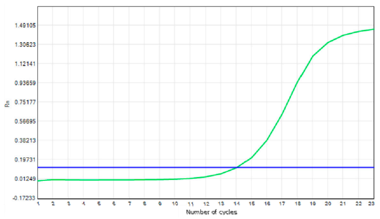 Number of cycles	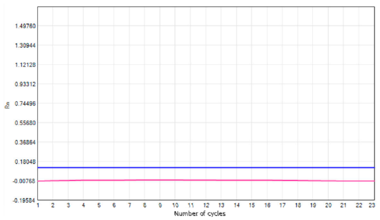 Number of cycles
**86**	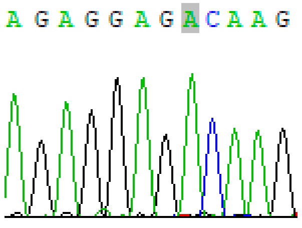 A G A G G A G A C A A G	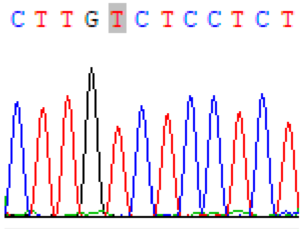 C T T G T C T C C T C T	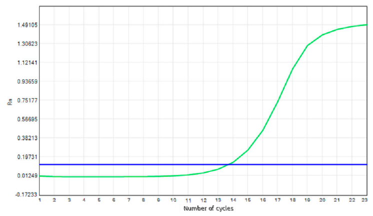 Number of cycles	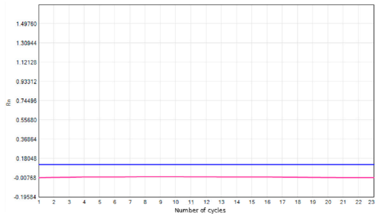 Number of cycles
**87**	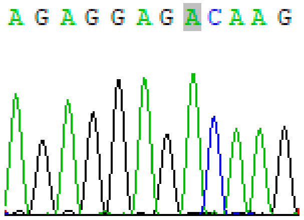 A G A G G A G A C A A G	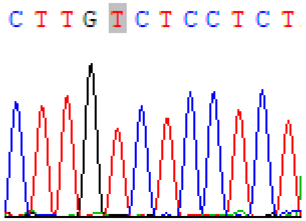 C T T G T C T C C T C T	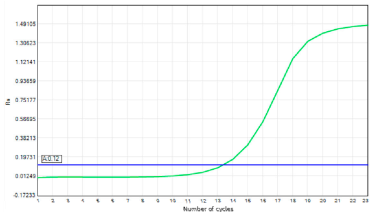 Number of cycles	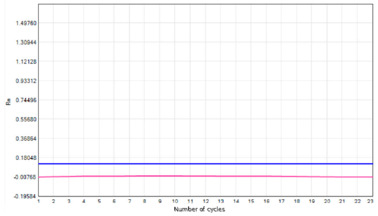 Number of cycles
**88**	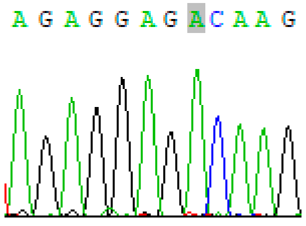 A G A G G A G A C A A G	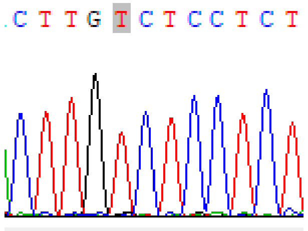 C T T G T C T C C T C T	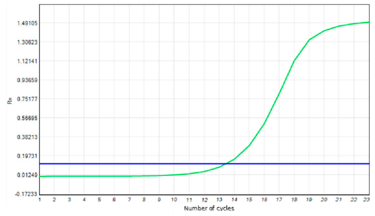 Number of cycles	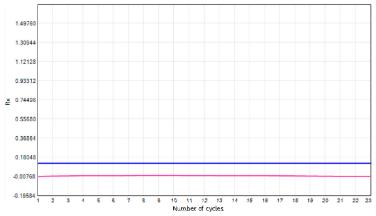 Number of cycles
**89**	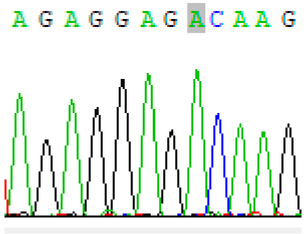 A G A G G A G A C A A G	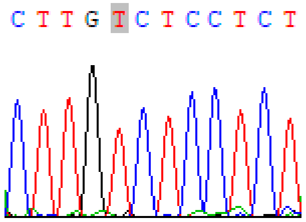 C T T G T C T C C T C T	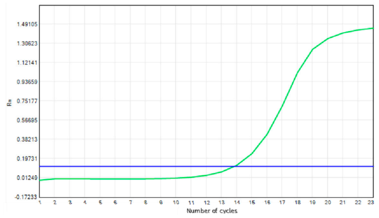 Number of cycles	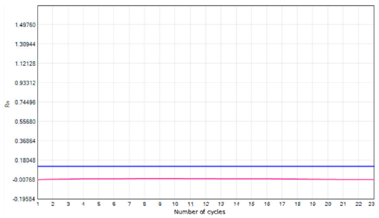 Number of cycles

**90**	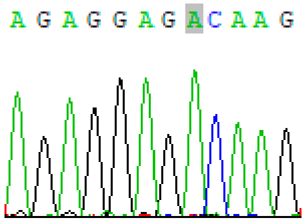A G A G G A G A C A A G	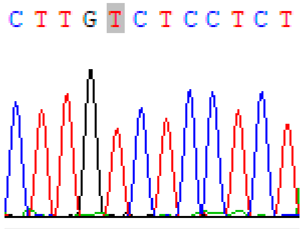C T T G T C T C C T C T	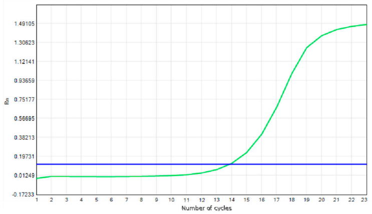Number of cycles	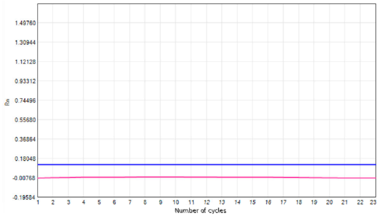Number of cycles
**91**	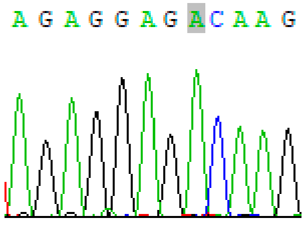A G A G G A G A C A A G	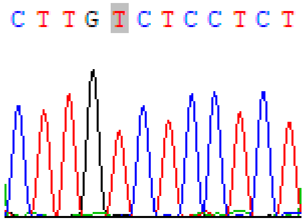C T T G T C T C C T C T	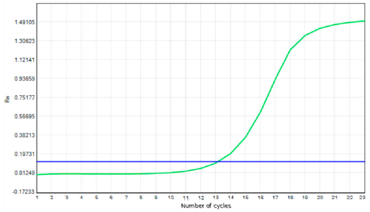Number of cycles	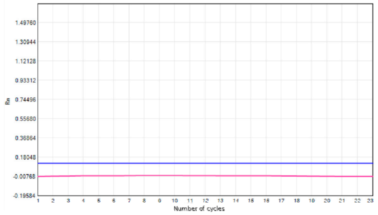Number of cycles
**92**	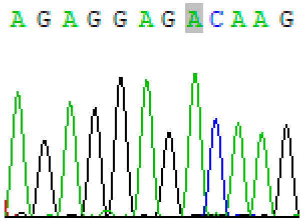A G A G G A G A C A A G	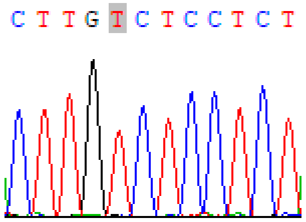C T T G T C T C C T C T	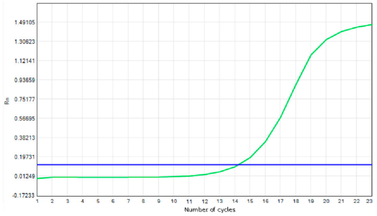Number of cycles	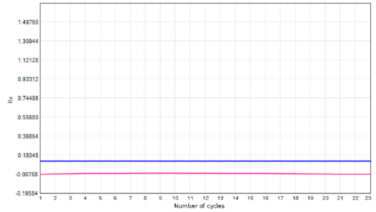Number of cycles
**93**	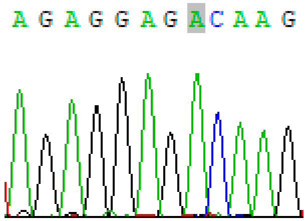A G A G G A G A C A A G	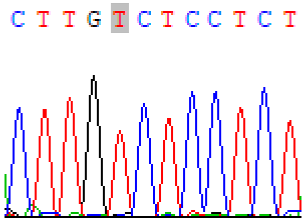C T T G T C T C C T C T	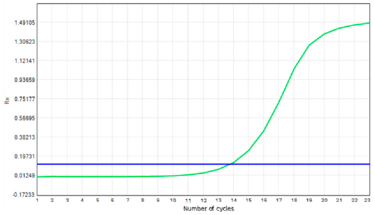Number of cycles	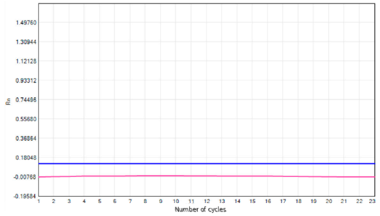Number of cycles
**94**	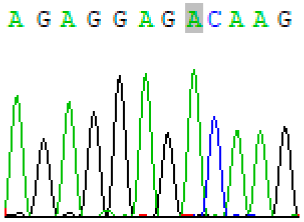A G A G G A G A C A A G	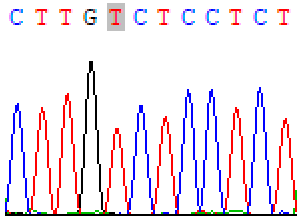C T T G T C T C C T C T	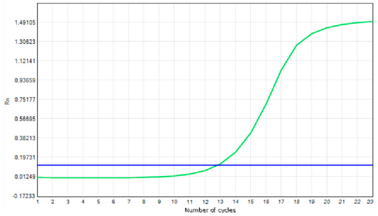Number of cycles	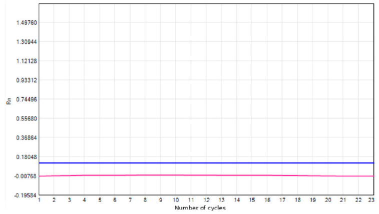Number of cycles

**95**	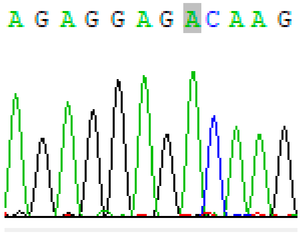A G A G G A G A C A A G	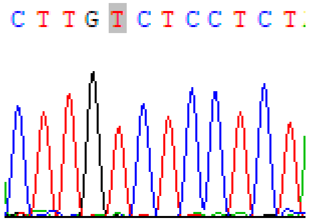C T T G T C T C C T C T	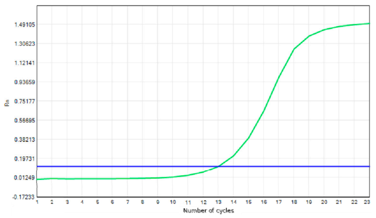Number of cycles	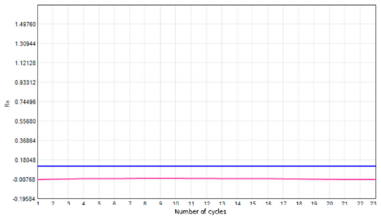Number of cycles
**96**	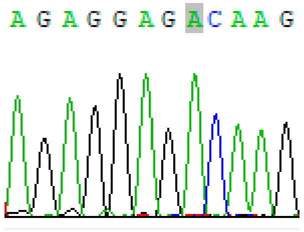A G A G G A G A C A A G	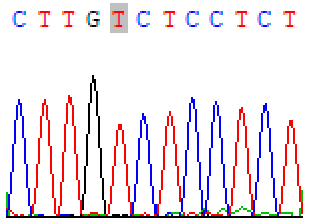C T T G T C T C C T C T	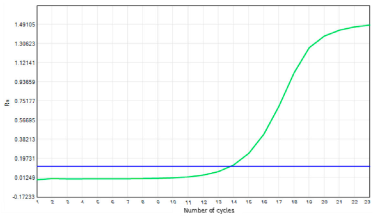Number of cycles	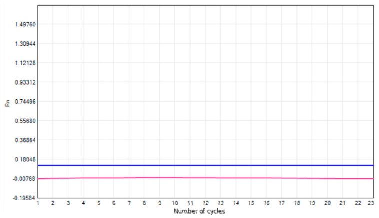Number of cycles
**97**	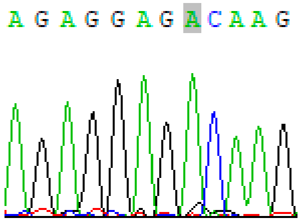A G A G G A G A C A A G	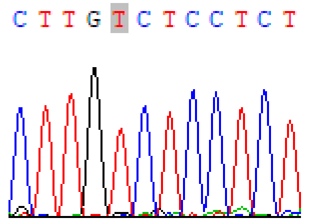C T T G T C T C C T C T	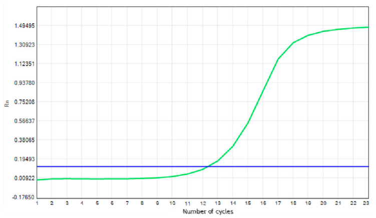Number of cycles	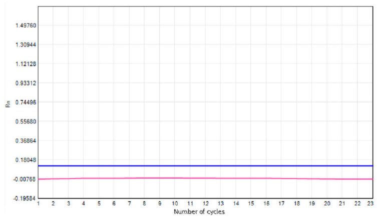Number of cycles
**98**	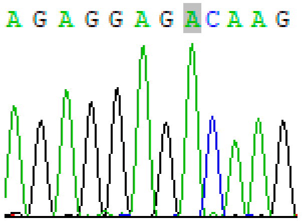A G A G G A G A C A A G	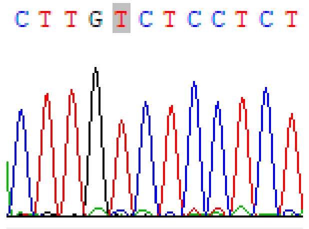C T T G T C T C C T C T	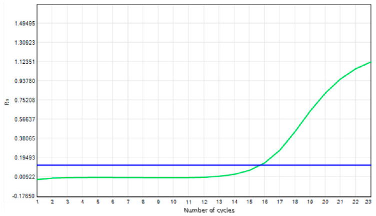Number of cycles	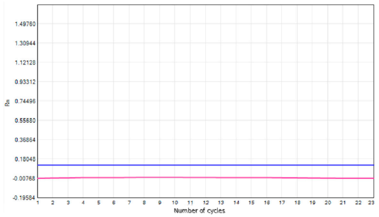Number of cycles
**99**	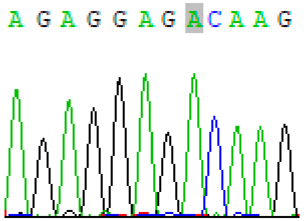A G A G G A G A C A A G	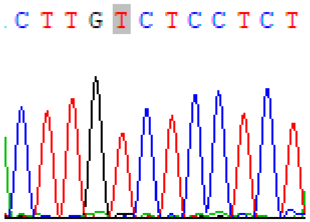C T T G T C T C C T C T	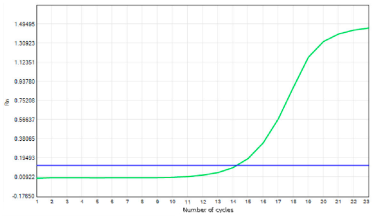Number of cycles	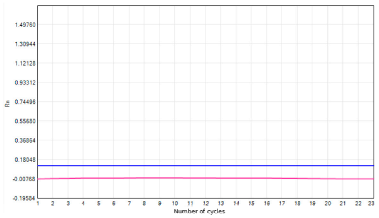Number of cycles

**100**	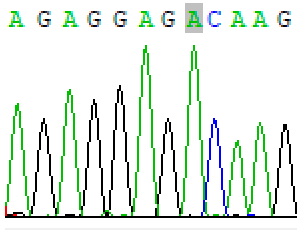	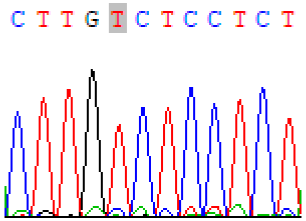	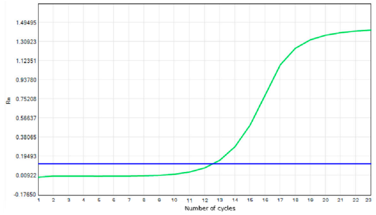	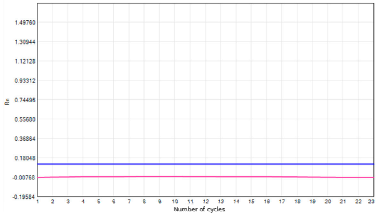
**101**	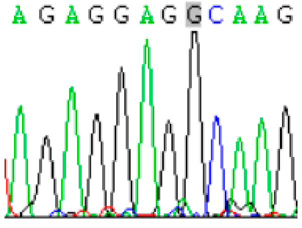	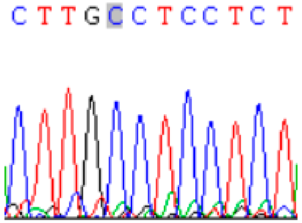	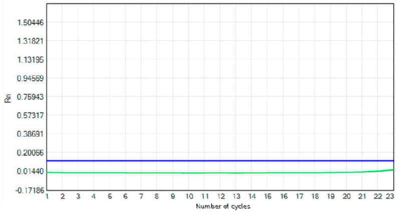	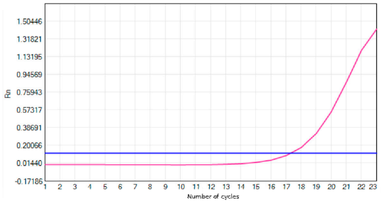
**102**	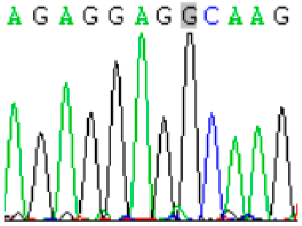	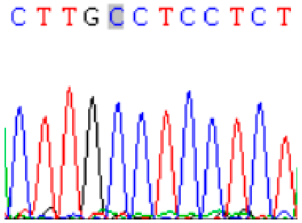	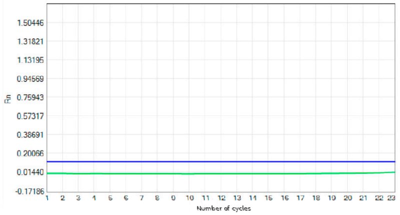	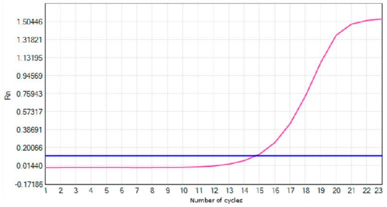
**103**	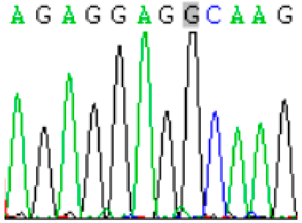	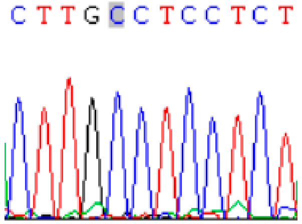	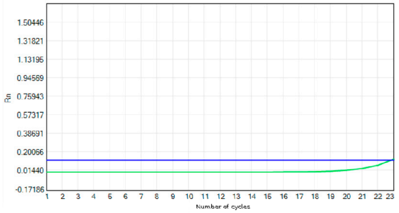	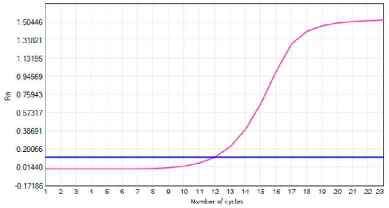
**104**	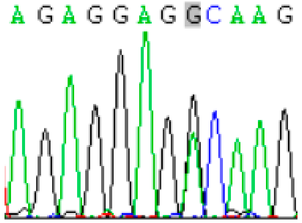	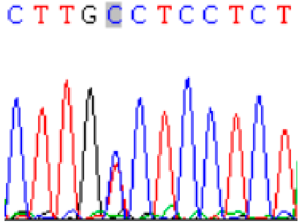	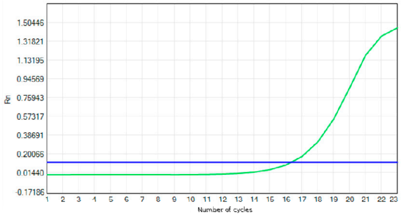	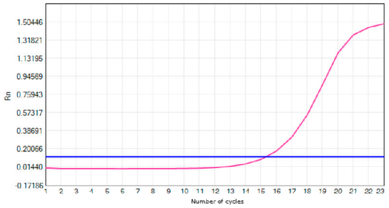

**105**	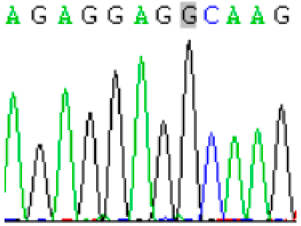	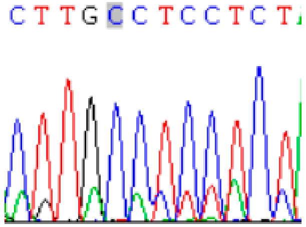	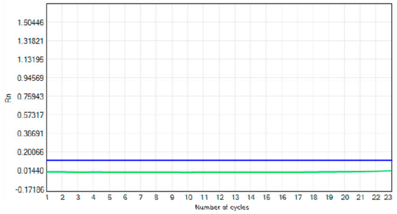	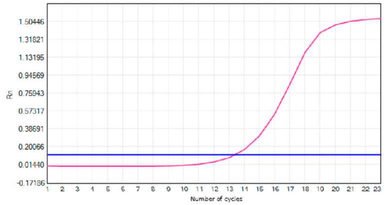
**106**	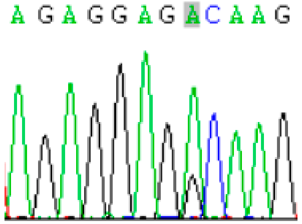	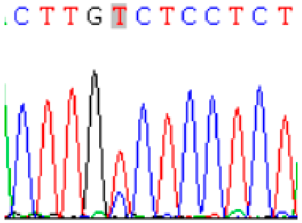	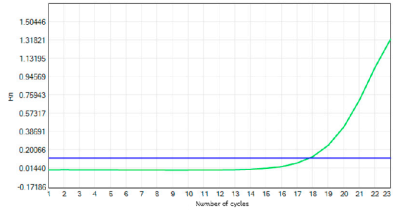	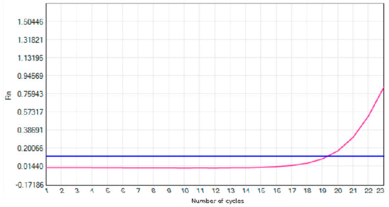
**107**	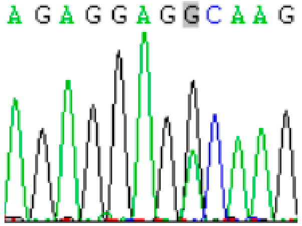	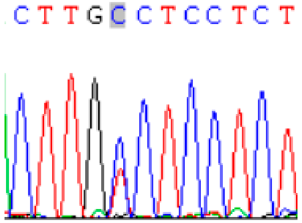	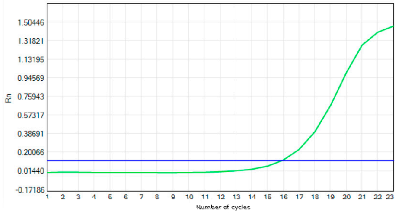	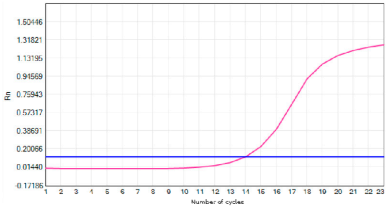
**108**	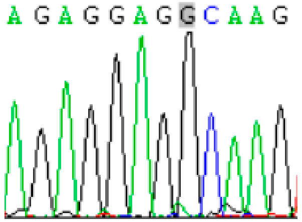	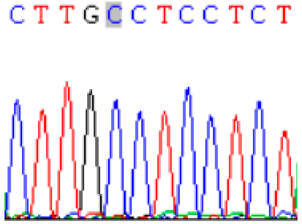	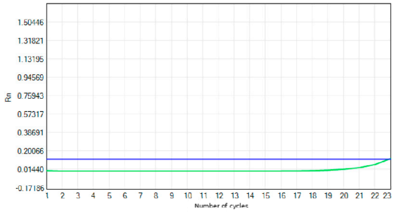	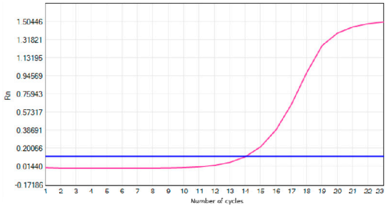
**109**	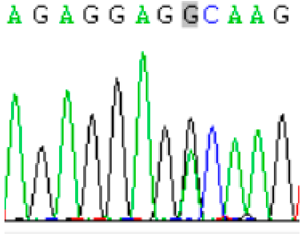	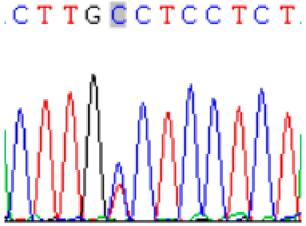	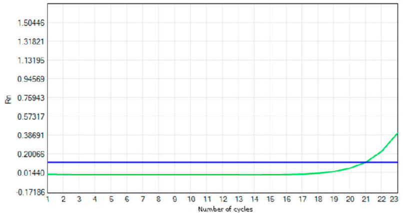	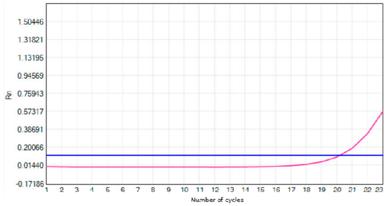

**110**	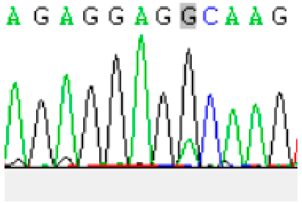	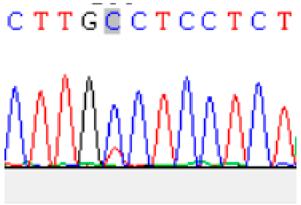	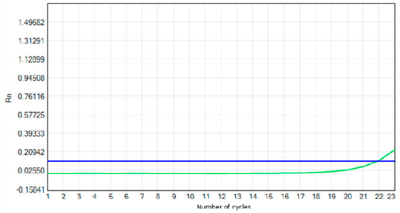	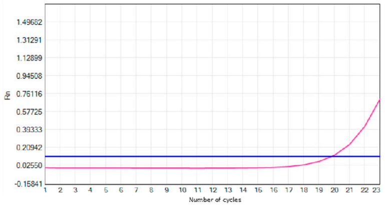
**111**	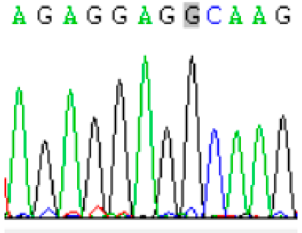	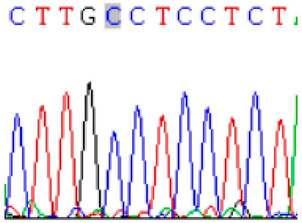	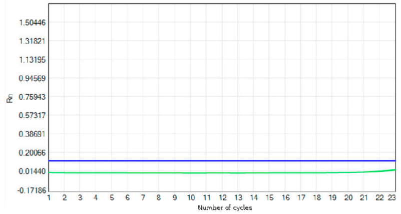	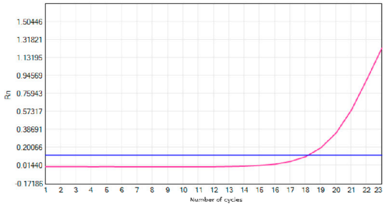
**112**	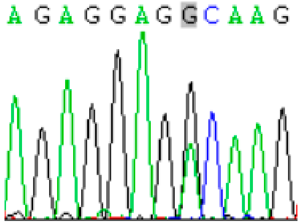	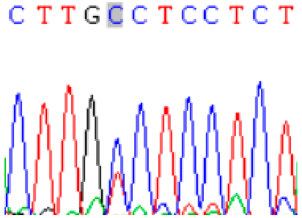	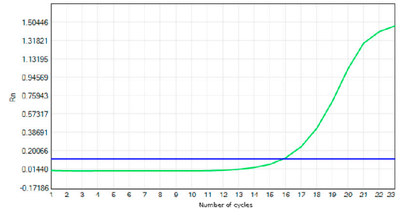	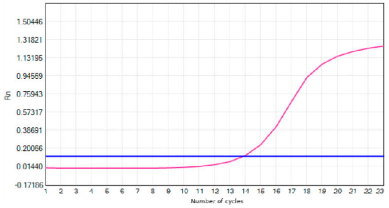
**113**	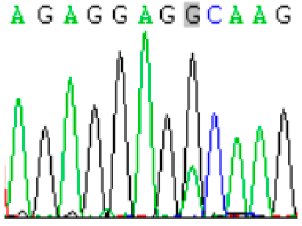	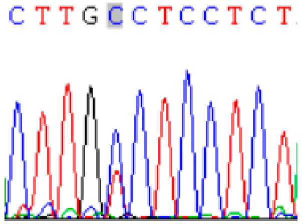	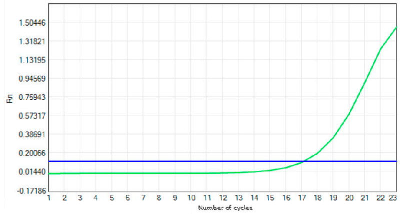	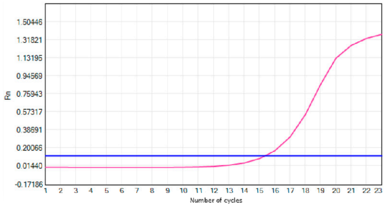
