# Supplementary material for: Application of the TaqMan ARMS-PCR Approach for Genotyping Drug-Induced Hearing Loss Using Dried Blood Samples
Source: Curr Issues Mol Biol. 2024 May 29;46(6):0. doi: 10.3390/cimb46060326 (PMC13176787; doi:10.3390/cimb46060326)
Supplement: Supplementary file 1 [file cimb-46-00326-s001.zip › Title of tabel and figure in Supplementary data.pdf]

Figure S1: Electropherograms of BamHI/SalI digestion. A: 1555A plasmid, B: 1555G plasmid (TIF);

Figure S2: Sanger sequencing plots. A: 1555A plasmid, B: 1555G plasmid (TIF);

Figure S3: Ct value of detection system with various wild/mutant plasmid ratio (n = 3). A: 1555A detection system, B: 1555G detection system (TIF);

Figure S4: Amplification plots of 1555A/G plasmid (n = 3). A: 1555A detection system,B: 1555G detection system (TIF);

Figure S5: Amplification plots of serial dilution wild and mutant plasmids (n = 3). A: 1555A detection system, B: 1555G detection system (TIF);

Figure S6: Amplification plots of serial dilution wild-type and mutant-type DBS DNA samples (n = 3). A: 1555A detection system, B: 1555G detection system (TIF);

Table S1: Plasmid sequences for the 1555A and 1555G genotypes (DOCX);

Table S2: Gene sequences of the 1555A and 1555G genotypes (DOCX);

Table S3: Detection of DBS DNA samples by ARMS-PCR and sanger sequencing (DOCX).
