# Supplementary figures and images for: Application of the TaqMan ARMS-PCR Approach for Genotyping Drug-Induced Hearing Loss Using Dried Blood Samples
Source: Curr Issues Mol Biol. 2024 May 29;46(6):0. doi: 10.3390/cimb46060326 (PMC13176787; doi:10.3390/cimb46060326)

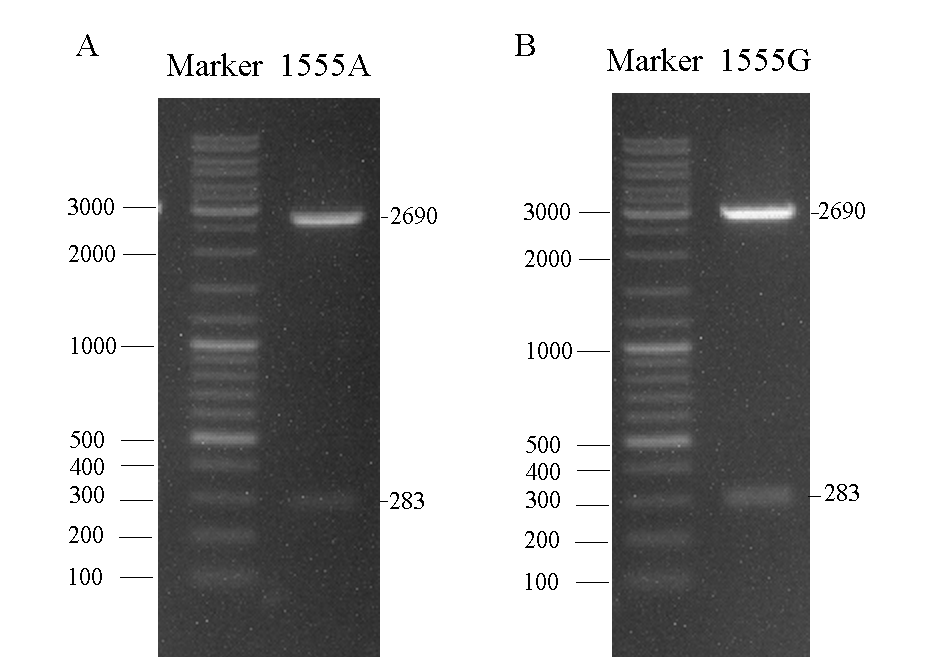

Supplement: Supplementary file 1 [file cimb-46-00326-s001.zip › Figure S1.tif]

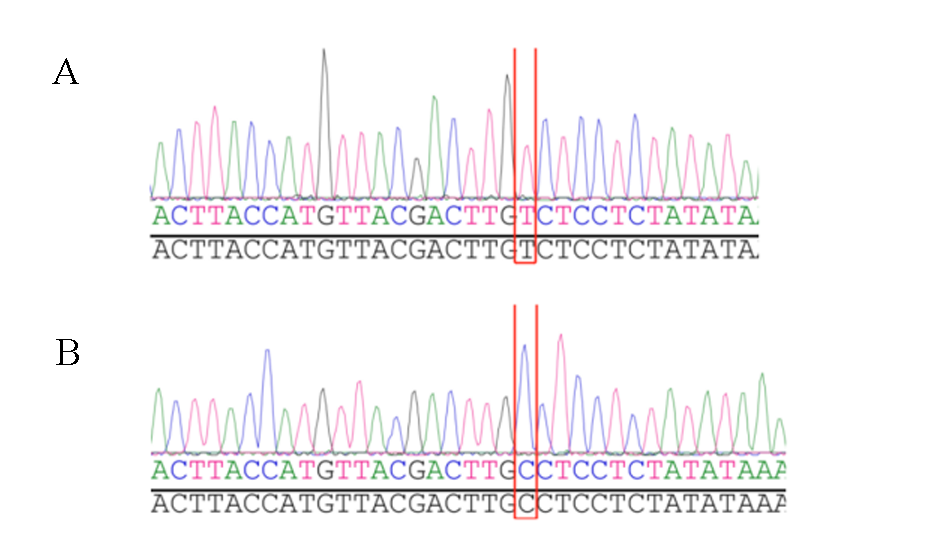

Supplement: Supplementary file 1 [file cimb-46-00326-s001.zip › Figure S2.tif]

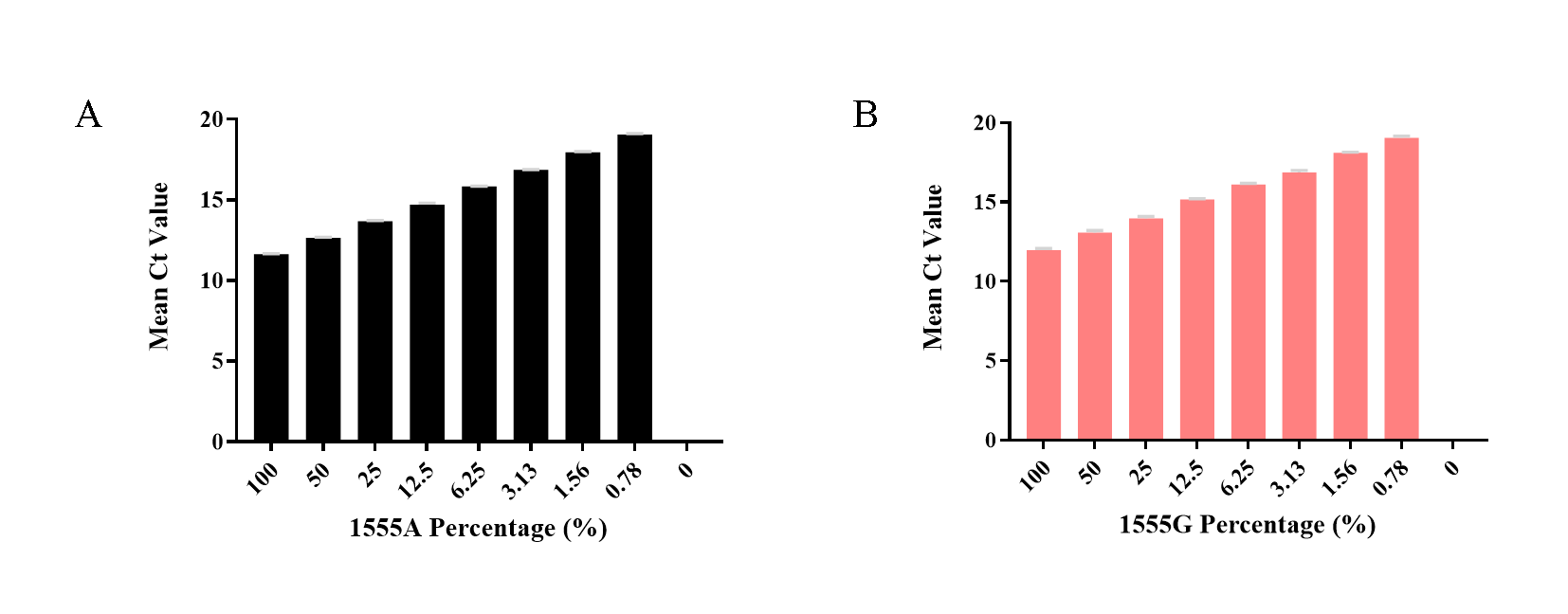

Supplement: Supplementary file 1 [file cimb-46-00326-s001.zip › Figure S3.tif]

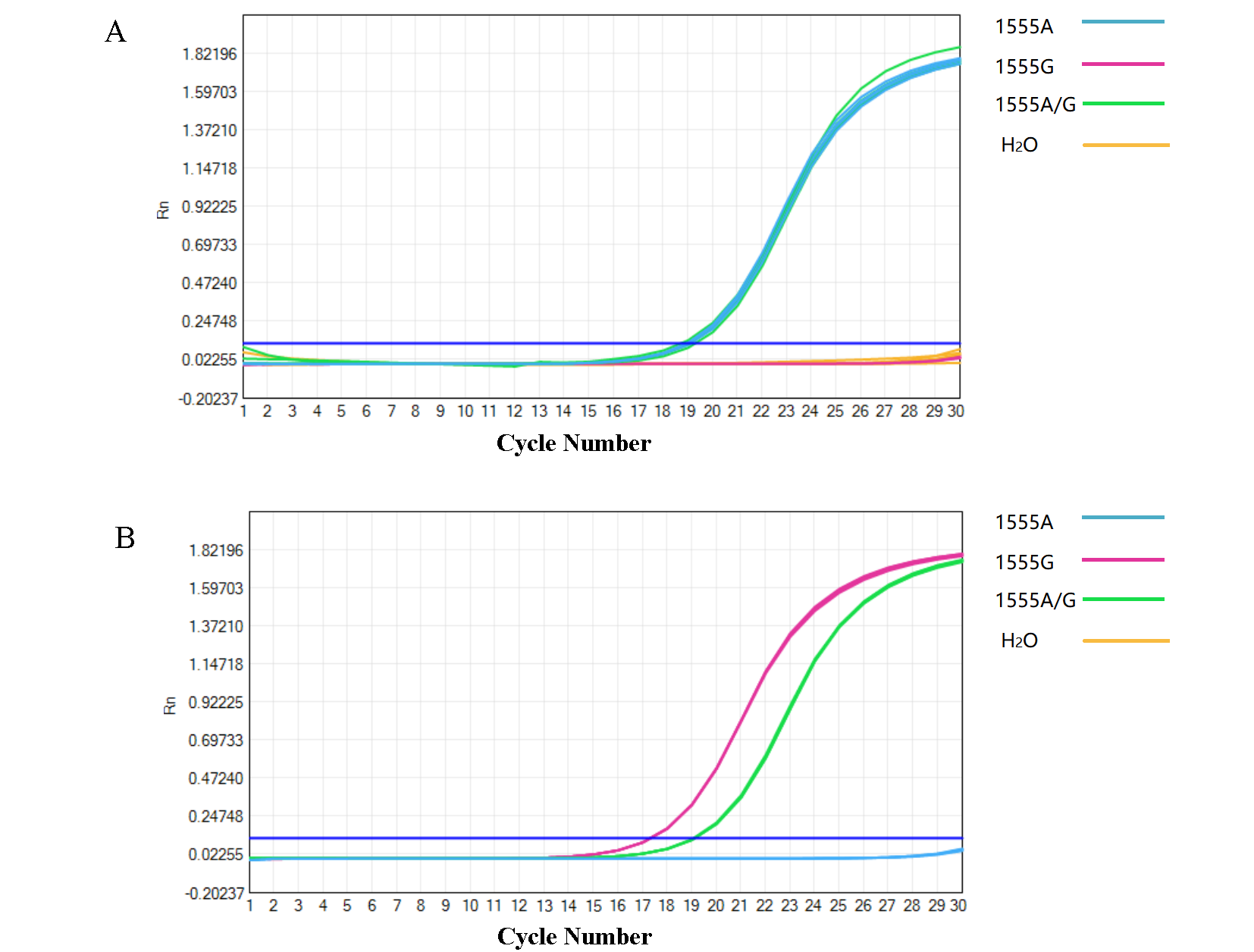

Supplement: Supplementary file 1 [file cimb-46-00326-s001.zip › Figure S4.tif]

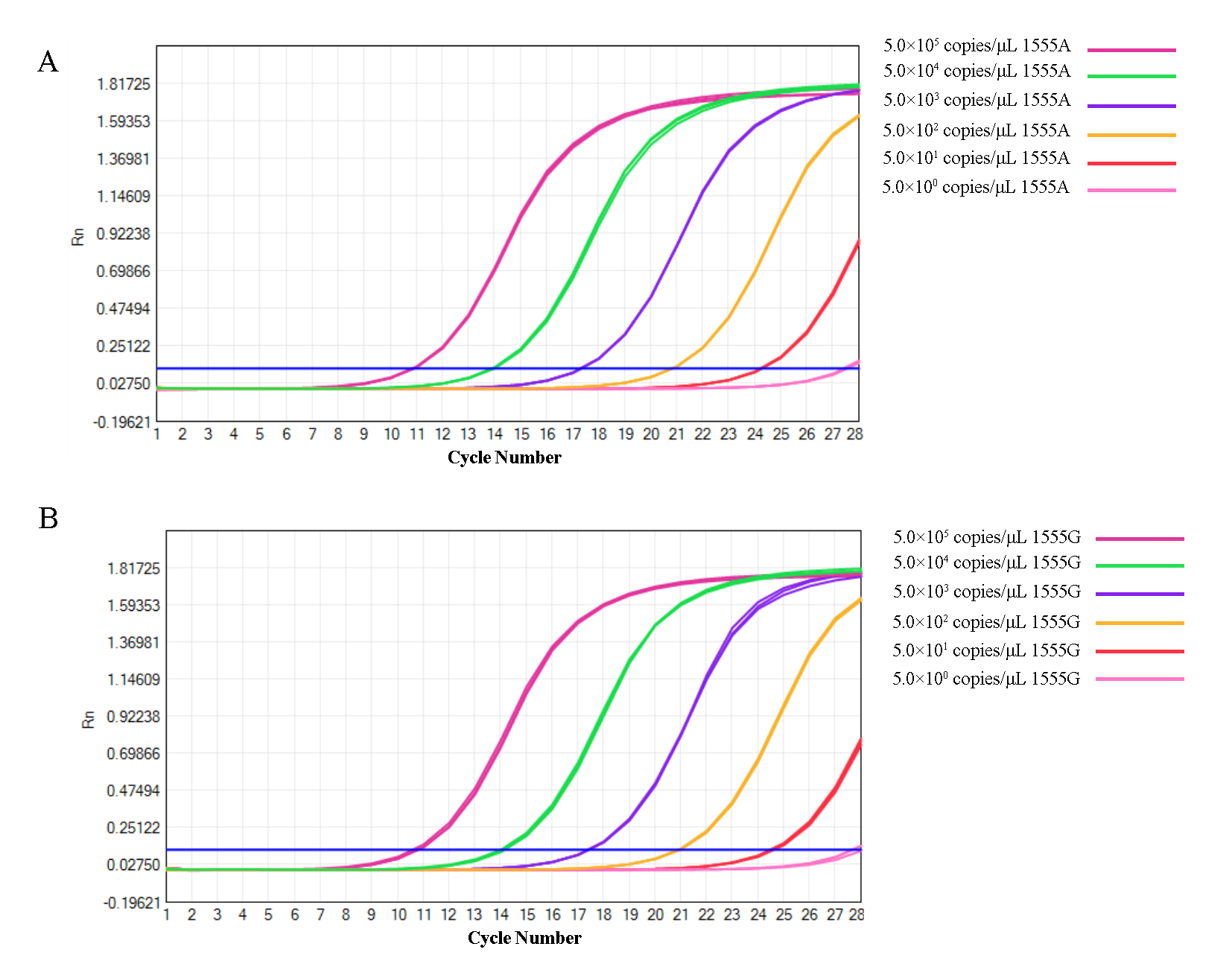

Supplement: Supplementary file 1 [file cimb-46-00326-s001.zip › Figure S5.tif]

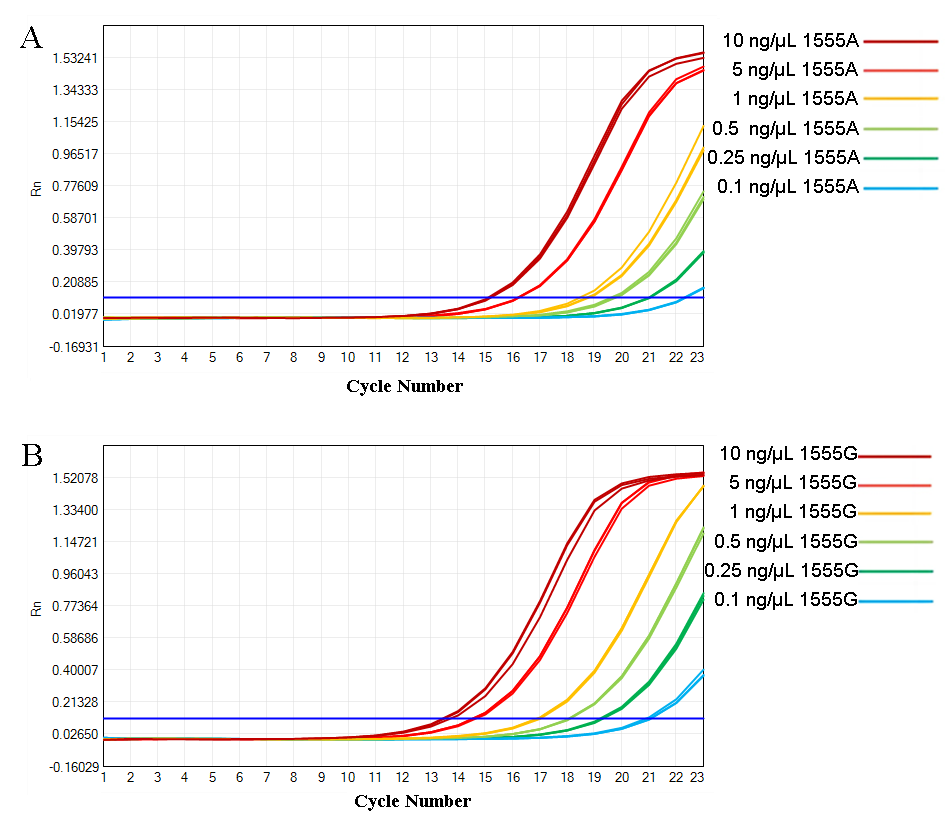

Supplement: Supplementary file 1 [file cimb-46-00326-s001.zip › Figure S6.tif]
